# A multi-omics insight on the interplay between iron deficiency and N forms in tomato

**DOI:** 10.3389/fpls.2024.1408141

**Published:** 2024-10-16

**Authors:** Arianna Lodovici, Sara Buoso, Begoña Miras-Moreno, Luigi Lucini, Nicola Tomasi, Pascual García-Pérez, Roberto Pinton, Laura Zanin

**Affiliations:** ^1^ Department of Agricultural. Food, Environmental and Animal Sciences, University of Udine, Udine, Italy; ^2^ Department for Sustainable Food Process, Università Cattolica del Sacro Cuore, Piacenza, Italy; ^3^ Department of Plant Biology, University of Murcia, Murcia, Spain

**Keywords:** ammonium, metabolomic, multi-omic, nitrate, nutrient interplay, root uptake, Strategy I, urea

## Abstract

**Introduction:**

Nitrogen (N) and iron (Fe) are involved in several biochemical processes in living organisms, and their limited bioavailability is a strong constraint for plant growth and yield. This work investigated the interplay between Fe and N nutritional pathways in tomato plants kept under N and Fe deficiency and then resupplied with Fe and N (as nitrate, ammonium, or urea) through a physiological, metabolomics and gene expression study.

**Results:**

After 24 hours of Fe resupply, the Fe concentration in Fe-deficient roots was dependent on the applied N form (following the pattern: nitrate > urea > ammonium > Fe-deficient control), and whereas in leaves of urea treated plants the Fe concentration was lower in comparison to the other N forms. Untargeted metabolomics pointed out distinctive modulations of plant metabolism in a treatment-dependent manner. Overall, N-containing metabolites were affected by the treatments in both leaves and roots, while N form significantly shaped the phytohormone profile. Moreover, the simultaneous application of Fe with N to Fe-deficient plants elicited secondary metabolites’ accumulation, such as phenylpropanoids, depending on the applied N form (mainly by urea, followed by nitrate and ammonium). After 4 hours of treatment, ammonium- and urea-treated roots showed a reduction of enzymatic activity of Fe(III)-chelate reductase (FCR), compared to nitrate or N-depleted plants (maintained in Fe deficiency, where FCR was maintained at high levels). The response of nitrate-treated plants leads to the improvement of Fe concentration in tomato roots and the increase of Fe(II) transporter (IRT1) gene expression in tomato roots.

**Conclusions:**

Our results strengthen and improve the understanding about the interaction between N and Fe nutritional pathways, thinning the current knowledge gap.

## Introduction

Despite being abundant on the Earth’s crust, Fe bioavailability under well-aerated and calcareous soil is strongly limited by a scarce solubility of ferric and ferrous forms in solution. It has been estimated that this unfavorable condition affects more than 30% of cultivated soils (Marastoni et al., 2020). Fe is acquired by crops with two distinct strategies based upon type of plant family (Strategy I used by all higher plants such as tomato except graminaceous, Strategy II used by graminaceous plants, Kobayashi et al., 2019; Mori, 1999). Strategy I, consist primarily in proton extrusion by activation of protonic pumps (H+-ATPase family; *AHA2* in Arabidopsis, *SlHA4* in tomato; Harper et al., 1990; Liu et al., 2016), followed by Fe(III) reduction to Fe(II) by FCR at the root surface level and then adsorbed into the root by iron regulated transporter (IRT; IRT1 in Arabidopsis; *SlIRT* 1 in tomato; Eide et al., 1996; Eckhardt et al., 2001).

Under Fe deficiency, crops exhibit leaf chlorosis and decreased photosynthesis, resulting in adverse consequences for yield and quality (Mahender et al., 2019; Zhang et al., 2019; Zuo and Zhang, 2011). In tomato roots, Zamboni et al. (2012) provided evidence that Fe deficiency induced the modulation of 97 genes in comparison to Fe sufficiency and, at the transcriptional level, this response is similar to the response activated by Arabidopsis (the so-called “Ferrome” by Schmidt and Buckhout, 2011).

Nowadays, one solution involves providing Fe to the soil in the form of synthetic chelates, causing a significant environmental and economic impact (Piccinelli et al., 2022). To move towards a more precise 4.0 agriculture, which is more restrained in the use of synthetic inputs, it is urgent to identify new agricultural practices aimed at the preservation of the environment and optimizing the already available soil resources (Anas et al., 2020; Congreves et al., 2021).

To identify new environmentally friendly solutions aimed at improving the efficiency of applied fertilizers and natural resources in the soil, the study of the Fe acquisition process in plants should consider the interplay of Fe with other nutrients (Fan et al., 2021). Among these, nitrogen (N) is the nutrient most used as fertilizer, mainly applied as urea, nitrate, or ammonium. More than 110 million tons of N fertilizers are used globally (FAOSTAT, 2021). Still, only a small quantity is effectively taken up by crops (30–40%), whereas the remaining fraction is lost in the environment (Eickhout et al., 2006; Gao et al., 2022; Sainju et al., 2019).

The influence of an N form on Fe acquisition is linked to changes occurring at the molecular and physiological levels in plants and the rhizosphere. Being nitrate and ammonium the ions with higher uptake (accounting for more than 70% of the total), their role in the control of cations and anions uptake, dry matter production, carbon assimilation rate, root apoplastic pH, and rhizosphere pH is significant (Arnozis and Findenegg, 1986; Marschner, 1995; Mengel, 1994). In particular, changes in the pH within the rhizosphere (e.g. alkalinization by nitrate or overall acidification by ammonium) and the plant apoplast largely modulate the uptake, remobilization and allocation of metals such as Fe and the acquisition of other nutrients (such as phosphorous, P; Thomson et al., 1993; Zou et al., 2001; Sarasketa et al., 2016; De la Peña et al., 2022). In addition to rhizospheric acidification and changes in plant metabolism, Zou et al. (2001) highlighted that ammonium even triggers nitric oxide production in *Arabidopsis*. This signaling molecule induces FCR activity and also the Fe release from the cell wall (especially from hemicellulose). Moreover, ammonium upregulates genes involved in Fe translocation from roots to shoots, such as *FRD3* and *NAS1* (*NA SYNTHASE1*), increasing the amount of soluble Fe in shoots and thus alleviating Fe deficiency symptoms in *Arabidopsis* (a reduction in interveinal leaf chlorosis; Zhu et al., 2019).

On the other hand, nitrate can lead to the alkalinization of the rhizosphere decreasing Fe solubility and of the root apoplast inhibiting the activity of FCR (Nikolic and Römheld, 2003), similar results were also shown at the leaf apoplast (Kosegarten et al., 1999). Moreover, Fe is a cofactor of several enzymes involved in the reductive assimilatory pathway of nitrate, such as nitrate reductase (NR), nitrite reductase (NiR) and glutamate synthase (GOGAT; Marschner, 1995). Thus, under Fe deficiency, nitrate assimilation is slowed down in plants (Alcaraz et al., 1986; Borlotti et al., 2012) and triggers a limitation in net nitrate uptake into roots at the same time (Iacuzzo et al., 2011). In apple, Sun et al. (2021) provided evidence that Fe deficiency symptoms were alleviated by a low nitrate nutrition, which in roots induced the accumulation of citrate and abscisic acid and activated their biosynthetic pathways, maintaining Fe homeostasis. This aspect is highly relevant for crop nutrition, especially in aerobic soils, where oxidation reactions convert N into nitrate, making this latter the main N-form available for plant nutrition.

Another important form of N in agriculture is urea, the most used fertilizer. In the last decade, the molecular mechanisms underlying urea uptake in cultivated plants started to be revealed (Wang et al., 2012a; Zanin et al., 2014, 2015); however, no information regarding the interaction between urea and the response to plant Fe deficiency is currently available.

Based on these considerations, there is still a gap in knowledge about the interplay between N and Fe nutritional pathways in plants (Nikolic et al., 2007; Borlotti et al., 2012; Liu et al., 2015; Chen et al., 2021), especially regarding the effects of different N forms in overcoming low Fe-bioavailability (Zou et al., 2001). Given the key role played by Fe in N assimilation and *vice versa*, strong cross-connections between the N and Fe nutritional pathways and the close relationships in the regulation and activation of their reciprocal acquisition mechanisms are likely (Liu et al., 2015; Chen et al., 2021). Studying the relationship between Fe and N in crop production is crucial as these two nutrients are fundamental to plant growth and productivity. A proper balance between Fe and N supply ensures that plants can efficiently gather energy and develop properly, leading to higher yields and better yield quality. Understanding this relationship helps in optimizing fertilizer use, preventing nutrient imbalances, and also improving soil fertility management, all of which are key to sustainable agricultural practices.

The present study aimed to evaluate the interplay between Fe and N nutritional pathway in tomato depending on the N-form applied: nitrate, urea or ammonium (the three most applied forms as N fertilizers). The characterization of plant responses to the Fe and N interplay will greatly advance our understanding of the role played by known and unknown components involved in Fe and N nutritional responses.

## Materials and methods

### Plant growth

*Solanum lycopersicum* L. cv “Marmande” (DOTTO Spa. Italy) were germinated for 7 days on filter paper moistened with 0.5 mM CaSO_4_ and then 180 seedlings were grown under hydroponic conditions as previously described by Tomasi et al. (2009). Twenty-one-day-old plants were then transferred to a Fe-free nutrient solution containing (mM): 0.70 K_2_SO_4_, 0.1 KCl, 2.00 Ca(NO_3_)_2_, 0.50 MgSO_4_, 0.10 KH_2_PO_4_; (µM): 10.00 H_3_BO_3_, 0.50 MnSO_4_, 0.50 ZnSO_4_, 0.20 CuSO_4_, 0.07 Na_2_MoO_4_ adjusted to pH 6.0 with KOH 1 M. After 14 days some tomato plants (35-day-old plants) were transferred to a N-free and Fe-free nutrient solution (-N-Fe NS, mM: 0.7 K_2_SO_4_, 0.1 KCl, 1.0 CaSO_4_, 0.5 MgSO_4_, 0.1 KH_2_PO_4_; µM: 10.00 H_3_BO_3_, 0.50 MnSO_4_, 0.50 ZnSO_4_, 0.20 CuSO_4_, 0.07 Na_2_MoO_4_). Under these nutritional conditions, plants were grown for 7 days, and the pH was buffered using 1.5 mM MES-BTP (pH 6.0). The nutrient solutions were renewed every 3 days. At the end of the growing period (42-day-old), plants were treated for 24 hours with different N-forms and Fe-resupply, resulting in a total of four experimental conditions: plants were transferred to -N-Fe NS with addition of 2 mM total N (in the form of nitrate, KNO_3_; urea, CO(NH_2_)_2_; or ammonium, (NH_4_)_2_SO_4_) and 5µM Fe-EDTA (*-Fe/+Fe+Nit*; *-Fe/+Fe+U*; *-Fe/+Fe+A* plants, respectively), or control plants were maintained in -N-Fe NS (without adding N and without Fe, *-Fe/-Fe-N* plants). As an additional control, 36 plants (35-day-old plants) were transferred to -N-Fe NS where was added 0.1 mM Fe-EDTA and maintained under this condition up to the end of the experiment (43-day-old; *+Fe/+Fe-N* plants; see **Supplementary Table S1**). The characterization of the early response of tomato plants to the 24-hour application of the same three N forms under Fe sufficiency has been previously studied and reported in Lodovici et al. (2024).

At the end of the experiment, tomato plants (43-day-old) were washed in deionized water and then roots, young leaves (YL, at 43 days were considered as YL the last fully expanded leaves) or old leaves (OL, at 43 days were considered as OL the first leaves above the cotyledons) were sampled for transcriptional, elemental content and metabolomic analyses.

During the whole growing period, the controlled climatic conditions were the following: 16/8 (day/night) photoperiod; 220 µmol m^-2^ s^-1^ light intensity; 25/20°C (day/night) temperature and 70-80% relative humidity. The light transmittance of leaves was monitored using the SPAD instrument (SPAD-502, Minolta, Osaka, Japan).

### Elemental analyses

The element concentrations of macro- and micro-nutrients in tomato samples were determined by Inductively Coupled Plasma–Optical Emission Spectroscopy (ICP-OES 5800, Agilent Technologies, Santa Clara, USA) and CHN analyzer (CHN IRMS Isoprime 100 Stable Isotope Ratio Mass Spectrometer, Elementar, Como, Italy).

For ICP-OES analyses, plant samples were oven-dried for 72 hours (at 60-80°C) and ground. For each sample, around 100 mg of ground powder was digested with concentrated ultrapure HNO_3_ using a microwave oven (ETHOS EASY, Milestone Srl, Sorisole (BG), Italy) accordingly to the USEPA 3052 method “Plant Xpress” (USEPA, 1995). Element quantifications were carried out using certified multi-element standards.

Regarding CHN analyses, plant shoots and roots were dried, and their total N and C contents were determined by CHN-IRMS (CHN IRMS Isoprime 100 Stable Isotope Ratio Mass Spectrometer, Elementar, Como, Italy).

### Metabolomic analysis

Roots, YL, and OL (four samples of each plant material per treatment) were ground in liquid nitrogen using a pestle and mortar. Briefly, 1.0 g of each plant sample was extracted in 10 mL of a hydroalcoholic solution (80:20 *v*/*v* methanol: water) acidified with 0.1% (*v/v*) formic acid, using an Ultra Turrax (Polytron PT, Switzerland). The extracts were then centrifuged (6000 × *g* for 10 min at 4°C) and the supernatants filtered through 0.22 μm cellulose syringe filters in UHPLC vials for analysis. The untargeted metabolomic analysis was performed using a quadrupole-time-of-flight mass spectrometer (6550 iFunnel, Agilent Technologies, Santa Clara, USA), coupled to an ultra-high-performance liquid chromatograph (UHPLC, 1290 series, Agilent Technologies, Santa Clara, USA) *via* a JetStream Electrospray ionization system, under previously optimized analytical conditions. Briefly, 6 μL of each sample were injected and a reverse-phase chromatographic separation was achieved by using a C18 column (Agilent Zorbax eclipse plus; 50 mm × 2.1 mm, 1.8 μm) and a water-acetonitrile binary gradient (from 6 to 94% organic in 32 min). The mass spectrometer worked in positive FULL SCAN mode (range 100 – 1200 *m/z*, 0.8 spectra/s, 30.000 FWHM). Compound identification was achieved through the ‘find-by-formula’ algorithm using the software Profinder B.07 (from Agilent Technologies) and the PlantCyc 9.6 database (Plant Metabolic Network, Filiz and Akbudak, 2020). The whole isotope pattern (i.e., monoisotopic mass, isotopic spacing, and isotopic ratio) was considered, considering 5 ppm for mass accuracy, resulting in a level 2 of confidence in annotation (Salek et al., 2013). The raw metabolomic dataset was extrapolated from the software Mass Profiler Professional B.12.06 (from Agilent Technologies) after post-acquisition data filtering (compounds do not present in 100% of the replications within at least one treatment were discarded), baselining and normalization.

### Ferric-chelate reductase activity

The FCR activity by tomato roots was determined according to Pinton et al., 1999. Briefly, the roots of single intact plants were incubated in the dark at 25°C for 30 min in 25 mL of an assay solution containing 0.5 mM CaSO_4_, 10 mM MES-KOH, 0.25 mM Fe(III)-EDTA, 0.5 mM Na_2_-bathophenanthrolinedisulfunic acid (BPDS). Every 15 min, the absorbance of the assay solution was measured at 535 nm. The amount of the Fe(III) reduced, as Fe(II)-BPDS_3_ complex, was calculated using an extinction coefficient of 22 mM^-1^ cm^-1^ and expressed as: µmol Fe(II) g^-1^ root FW h^-1^ (FW, Fresh Weight).

### Gene expression analysis

Tomato roots were ground in liquid nitrogen. Total RNA was extracted from approximately 60-70 mg of powder using the Spectrum Plant Total RNA Kit (Sigma Aldrich, St. Louis, MO, USA) according to the manufacturer’s instructions (protocol A). RNA quantity and quality were inspected through a NanoDrop device (NanoDrop Technologies, Wilmington, Delaware, USA) and by migration in agarose gel, respectively. Afterwards, 1 µg of extracted RNA was retrotranscribed into cDNA, adding: 1 µL of Oligo-d (T) 70 µM, 1 µl dNTP (10 mM), 20 U Rnase inhibitors, 200 U M-MLV Reverse Transcriptase (M-MLV Reverse Transcriptase Sigma Aldrich, St. Louis, MO, USA) according to the manufacturer’s instruction.

Using primer3 software (version 4.0.1) primers were designed and then synthesized by Merck (MERCK KGAA Darmstadt, Germany; **Supplementary Table S2**). RT-PCR analysis was performed with CFX96 Touch Real-Time PCR Detection System (Bio-Rad, Hercules, CA, USA). Data were referred to the averaged expression of two housekeeping genes *SlEF1* and *SlUbi* (**Supplementary Table S2**). Data were normalized using the 2^–ΔΔCT^ according to Livak and Schmittgen (2001). The efficiency of each set of primer was estimated using the qPCR package for statistical analysis by R software (R version 2.9.1. www.dr-spiess.de/qpcR.html) as indicated by Ritz and Spiess (2008; R Core Team, 2021).

### Statistical analysis

Three independent experiments were performed and a pool of roots of young leaves or old leaves from three tomato plants was used for each sample (Roots, YL and OL, respectively).

Statistical analyses were performed by SigmaPlot 14.0 (SigmaPlot Software, CA, USA), using one-way ANOVA with a Holm-Sidak’s test as *post hoc* test for multiple comparisons (p-value <0.05, N = 3).

The metabolomic dataset was processed as previously reported (García-Pérez et al., 2021). Outliers were detected and removed, and the remaining samples were employed for multivariate statistics and post-acquisition analyses. Hierarchical cluster analysis (HCA) (Euclidean distance, Ward’s linkage), one-way ANOVA and the subsequent fold-change (FC) analysis (*p* < 0.01, Bonferroni multiple testing correction; FC ≥ 2) were obtained from the Mass Profiler Professional B.12.06 software tool. The differential compounds were then interpreted using the PlantCyc Pathway Tool (Karp et al., 2010).

Moreover, the raw metabolomic dataset was exported into SIMCA 16 (Umetrics, Malmo, Sweden) for orthogonal projection to latent structures discriminant analysis (OPLS-DA) supervised modelling. Each model was cross-validated, inspected for outliers and overfitting, and then R^2^Y (goodness-of-fit) and Q^2^Y (goodness-of-prediction) parameters were recorded. Finally, the variables importance in projection (VIP) method allowed identifying discriminant compounds (VIP markers) with a VIP score > 1.3.

Regarding gene expression analysis and elemental content analyses the heatmap and principal component analyses (PCAs) were generated using ClustVis (https://biit.cs.ut.ee/clustvis/; Metsalu and Vilo, 2015) webtool using the fold parameters. The significance of the clustering observed in PCAs was assessed by PERMANOVA test using 5000 permutations performed with R version 4.3.0 (vegan package, Oksanen et al., 2014).

## Results

### Morphological observations

Morphometric measures were performed in all the considered plant organs (YL, OL, roots, and whole shoots (S)) under our experimental conditions. At the end of the growing period and after the 24-hour treatment, Fe-deficient tomato plants (-Fe/-Fe-N, -Fe/+Fe+Nit, -Fe/+Fe+U, -Fe/+Fe+A) resulted in being homogeneous at whole plants and foliar cover level (**Supplementary Figures S1**, **S2**). As expected, the SPAD values in YL were highly responsive to Fe nutritional status, as Fe-deficient plants displayed the lowest values, whereas the highest values were observed under Fe-sufficiency. After 24 hours, the resupply of nitrogen to Fe-deficient plants increased the SPAD values compared to the Fe-deficient control (-Fe/-Fe-N), increasing significantly under nitrate or ammonium nutrition. The SPAD values measured in old leaves were significantly lower in -Fe/-Fe-N, -Fe/+Fe+Nit and -Fe/+Fe+A if compared to +Fe/+Fe-N plants (**Supplementary Figure S3**). No significant changes in the dry biomass were detected in shoots and roots among treatments (**Supplementary Figure S3**). The height of shoots of +Fe/+Fe-N plants was significantly higher than those detected in plants grown under Fe deficiency (**Supplementary Figure S3**).

### Elemental content

**Figure 1:**
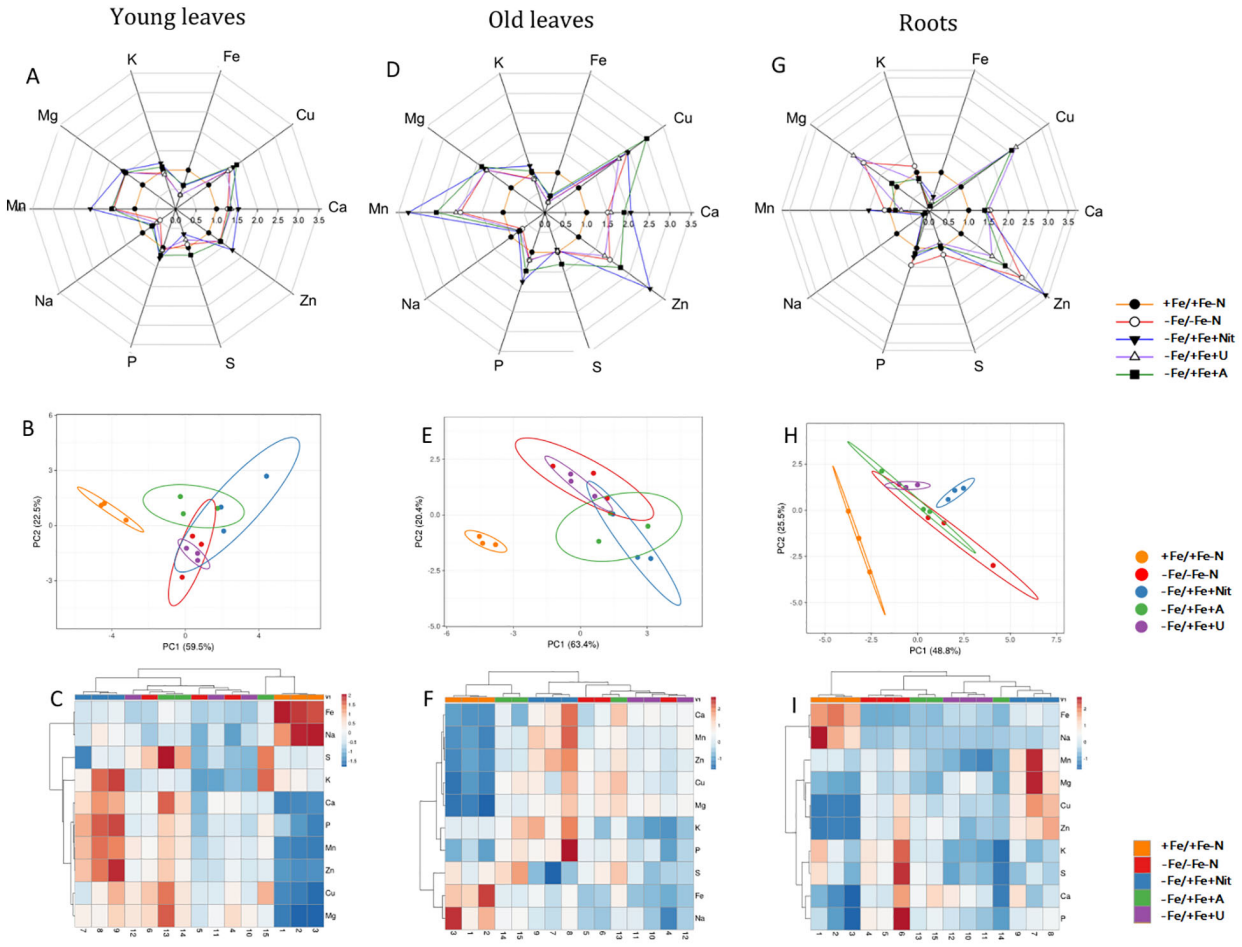
Elemental content of tomato plants after 24 hours of treatment with different N sources in old leaves, young leaves and roots. In radar plots, the concentration of considered elements in young leaves **(A)**, old leaves **(D)** and roots **(G)** was scaled to average value of +Fe/+Fe-N samples (value 1.0). PCA analyses show principal component 1 and principal component 2 that explain: 63.4% and 20.4% of the total variance in old leaves **(B)**, 59.5% and 22.5% of the total variance in young leaves **(E)** and 48.8% and 25.5% of the total variance in roots **(H)**. In heatmaps, a clustering of elemental concentration and samples in old leaves **(C)**, young leaves **(F)** and roots **(I)**.

After 24 hours of N and Fe resupply, the concentration of macro- and micro-nutrients in OL, YL and roots were determined (**Table 1**, **Supplementary Table S3**, **Figure 1**).

**Table 1:** Elemental concentration in tomato plants.

μg g^-1^ DW	Cu	Fe	Mn	Na	Zn
Young leaves					
+Fe/+Fe-N	12.6 ± 1^b^	196.2 ± 33.2^a^	32.7 ± 2.1^c^	208 ± 41.2^a^	31.5 ± 1.4^c^
-Fe/-Fe-N	20.7 ± 3^a^	69.6 ± 12^c^	49.8 ± 4.2^b^	98.1 ± 20.6^b^	42.4 ± 3.4^b^
-Fe/+Fe+Nit	22 ± 4.4^a^	115.5 ± 23.4^b^	68.3 ± 8.1^a^	137 ± 21.2^b^	54 ± 3.6^a^
-Fe/+Fe+U	19.8 ± 1^ab^	70.2 ± 4^c^	51.2 ± 4^b^	120 ± 16.3^b^	42.8 ± 1.1^b^
-Fe/+Fe+A	23.2 ± 2.2^a^	119.8 ± 17.8^b^	51.2 ± 5^bc^	150 ± 10.9^ab^	42.9 ± 5.3^b^
Old leaves					
+Fe/+Fe-N	8.4 ± 0.9^b^	253 ± 10.2^a^	33.1 ± 2^c^	458.1 ± 22.4^a^	26.7 ± 1.2^c^
-Fe/-Fe-N	20.4 ± 4.2^a^	65 ± 3^c^	67 ± 11.6^b^	304.2 ± 11.7^c^	51.4 ± 8.4^b^
-Fe/+Fe+Nit	20.6 ± 5^a^	94.1 ± 15.8^b^	108 ± 7.9^a^	359.1 ± 23.7^b^	83 ± 10.1^a^
-Fe/+Fe+U	18.4 ± 2.9^a^	63.8 ± 7.8^c^	70.5 ± 12.3^b^	339 ± 9.6^bc^	47.2 ± 5.5^b^
-Fe/+Fe+A	25.2 ± 3.2^a^	106 ± 14.1^b^	86 ± 9^ab^	341 ± 8.8^bc^	59.7 ± 11.2^b^
Roots					
+Fe/+Fe-N	121 ± 5.1^c^	1182 ± 110^a^	117 ± 10.6^b^	4339 ± 1333^a^	144 ± 5.21^c^
-Fe/-Fe-N	451 ± 108^ab^	58.9 ± 10.7^e^	130 ± 19.2^ab^	545 ± 184^b^	416 ± 116^a^
-Fe/+Fe+Nit	618 ± 97.7^a^	427 ± 104^b^	177 ± 35.7^a^	806 ± 154^b^	522 ± 60.7^a^
-Fe/+Fe+U	327 ± 41.4^b^	271 ± 42.5^c^	81.8 ± 9.5^b^	524 ± 20.8^b^	283 ± 61.0^b^
-Fe/+Fe+A	310 ± 50.2^b^	130 ± 28.8^d^	98.9 ± 9^b^	529 ± 33^b^	341 ± 77.1^abc^
mg g^-1^ DW	Ca	K	Mg	P	S
*Young leaves*					
+Fe/+Fe-N	11.1 ± 0.2^b^	40 ± 0.1^ab^	4.7 ± 0.2^b^	5.6 ± 0.2^b^	17.5 ± 1.8^ab^
-Fe/-Fe-N	14.1 ± 1^ab^	36.8 ± 4^b^	7.1 ± 0.3^a^	6 ± 0.5^ab^	16.1 ± 2.3^abc^
-Fe/+Fe+Nit	17 ± 2^a^	47.2 ± 3.8^a^	7.5 ± 0.5^a^	7.1 ± 0.9^a^	11.4 ± 2.3^c^
-Fe/+Fe+U	15.1 ± 2^ab^	35.3 ± 1.3^b^	7.2 ± 0.2^a^	5.9 ± 0.2^ab^	13.9 ± 1.4^c^
-Fe/+Fe+A	14.6 ± 2.7^ab^	43.4 ± 3.2^a^	7.1 ± 0.7^a^	6.7 ± 0.2^ab^	20.9 ± 1.1^a^
Old leaves					
+Fe/+Fe-N	16.9 ± 0.3^b^	39.8 ± 2.1^ab^	4.6 ± 0.2^b^	4.5 ± 0.5^c^	38.1 ± 0.8^ab^
-Fe/-Fe-N	25.4 ± 4.5^ab^	33.8 ± 2.9^b^	8 ± 1.2^a^	5.3 ± 0.5^c^	37.4 ± 6.9^ab^
-Fe/+Fe+Nit	34.7 ± 1.9^a^	46.8 ± 5.4^a^	8.2 ± 0.5^a^	7.8 ± 0.4^a^	35.6 ± 4.8^b^
-Fe/+Fe+U	26.7 ± 1.5^ab^	33 ± 1.9^b^	8 ± 0.3^a^	5.4 ± 0.2^c^	37 ± 4.2^ab^
-Fe/+Fe+A	32 ± 6.8^a^	41.5 ± 6.6^a^	8.6 ± 1.5^a^	6.6 ± 0.3^b^	49.6 ± 3.1^a^
Roots					
+Fe/+Fe-N	3.7 ± 0.5	62.5 ± 11.9^ab^	2.3 ± 0.4^b^	5.7 ± 0.8^b^	10.9 ± 1.6
-Fe/-Fe-N	5.7 ± 1.2	72.9 ± 9.8^a^	4.7 ± 1.2^b^	8.2 ± 1.3^a^	12.8 ± 1.8
-Fe/+Fe+Nit	5.2 ± 1	54.1 ± 5.4^ab^	10 ± 2.9^a^	7 ± 0.2^ab^	10 ± 0.5
-Fe/+Fe+U	5.5 ± 0.6	47.5 ± 0.7^b^	5.4 ± 1.3^b^	6.3 ± 0.2^ab^	9.4 ± 0.3
-Fe/+Fe+A	5.2 ± 1.5	51.9 ± 10.1^ab^	2.7 ± 0.5^b^	6.5 ± 0.8^ab^	10 ± 1.7
mg g^-1^ DW	C	N			
mg g^-1^ DW		C		N	
Shoots					
+Fe/+Fe-N		368 ± 8.2^a^		25.4 ± 1.0^ab^	
-Fe/-Fe-N		324 ± 13.9^ab^		20.4 ± 1.6^b^	
-Fe/+Fe+Nit		335 ± 11.8^ab^		30.6 ± 1.9^a^	
-Fe/+Fe+U		337 ± 5.8^ab^		22.2 ± 2.3^b^	
-Fe/+Fe+A		307 ± 36.2^b^		22.1 ± 4.2^b^	
Roots					
+Fe/+Fe-N		415 ± 2.8^a^		30.4 ± 1.8^b^	
-Fe/-Fe-N		407 ± 9.3^a^		31.8 ± 0.8^b^	
-Fe/+Fe+Nit		385.8 ± 3^b^		38.6 ± 1.0^a^	
-Fe/+Fe+U		399.4 ± 6.8^ab^		30.8 ± 1.5^b^	
-Fe/+Fe+A		401 ± 7.4^ab^		40.1 ± 0.4^a^	

Plants were maintained in N-free nutrient solution and Fe sufficiency (control +Fe/+Fe-N) or Fe deficiency (control -Fe/-Fe-N) or exposed to three different N sources and Fe-resupply (nitrate and Fe-EDTA, -Fe/+Fe+Nit; urea and Fe-EDTA, -Fe/+Fe+U; or ammonium and Fe-EDTA, -Fe/+Fe+A) for 24 hours. Data refers to the analyses performed on three plant organs: young leaves, old leaves and roots. Data refers to mean values ± SD; letters refer to statistical significance for each element and plant organ among experimental conditions (Holm–Sidak test ANOVA. N =3. p-value < 0.05). Data are expressed in μg g^-1^ or mg g^-1^ dry weight (DW).

The principal component analysis (PCA) showed that +Fe/+Fe-N was separated from the other treatments (-Fe/-Fe-N, -Fe/+Fe+Nit, -Fe/+Fe+U or -Fe/+Fe+A, which generally clustered together; PERMANOVA p-value<0.001 for YL and OL, p-value<0.05 for roots, **Figure 1**). **Supplementary Figure S4** reports the PCA of plants grown only under nitrogen and Fe deficiency.

The results mentioned above are reflected in the specific elemental concentration. The supply of nitrate or ammonium to N and Fe-deficient plants induced an increase of N concentration in shoots and roots, respectively, in comparison to the controls and urea-treated plants (-Fe/-Fe-N, +Fe/+Fe-N, -Fe/+Fe+U). It is interesting to highlight that Fe concentration is significantly and differentially concentrated considering each applied experimental condition. As expected, +Fe/+Fe-N has the highest Fe concentration, whereas the other conditions followed the pattern: -Fe/+Fe+Nit > -Fe/+Fe+U > -Fe/+Fe+A > -Fe/-Fe-N.

Regarding the other analyzed elements, in YL, the application of Nit as N-form resulted in a significant increase of Mn and Zn in comparison to +Fe/+Fe-N, -Fe/-Fe-N, -Fe/+Fe+U or -Fe/+Fe+A treatments. Besides, Fe concentration resulted in being higher in YL treated with Nit and A in comparison to -Fe/-Fe-N and -Fe/+Fe+U, while A supply led to a higher concentration of S in comparison to -Fe/+Fe+Nit and -Fe/+Fe+A treatment.

The elemental analysis in OL showed an increase in the concentration of Zn and P when Nit was supplied as N-form in comparison to +Fe/+Fe-N, -Fe/-Fe-N, -Fe/+Fe+U or -Fe/+Fe+A treatment. Moreover, as in YL, Fe concentration increased when Nit and A were supplied to the nutrient solution compared to -Fe/-Fe-N and U treatment.

In roots, the supply of Nit led to a higher concentration of Mn and Mg compared to the other treatments.

### Metabolomic analysis

**Figure 2:**
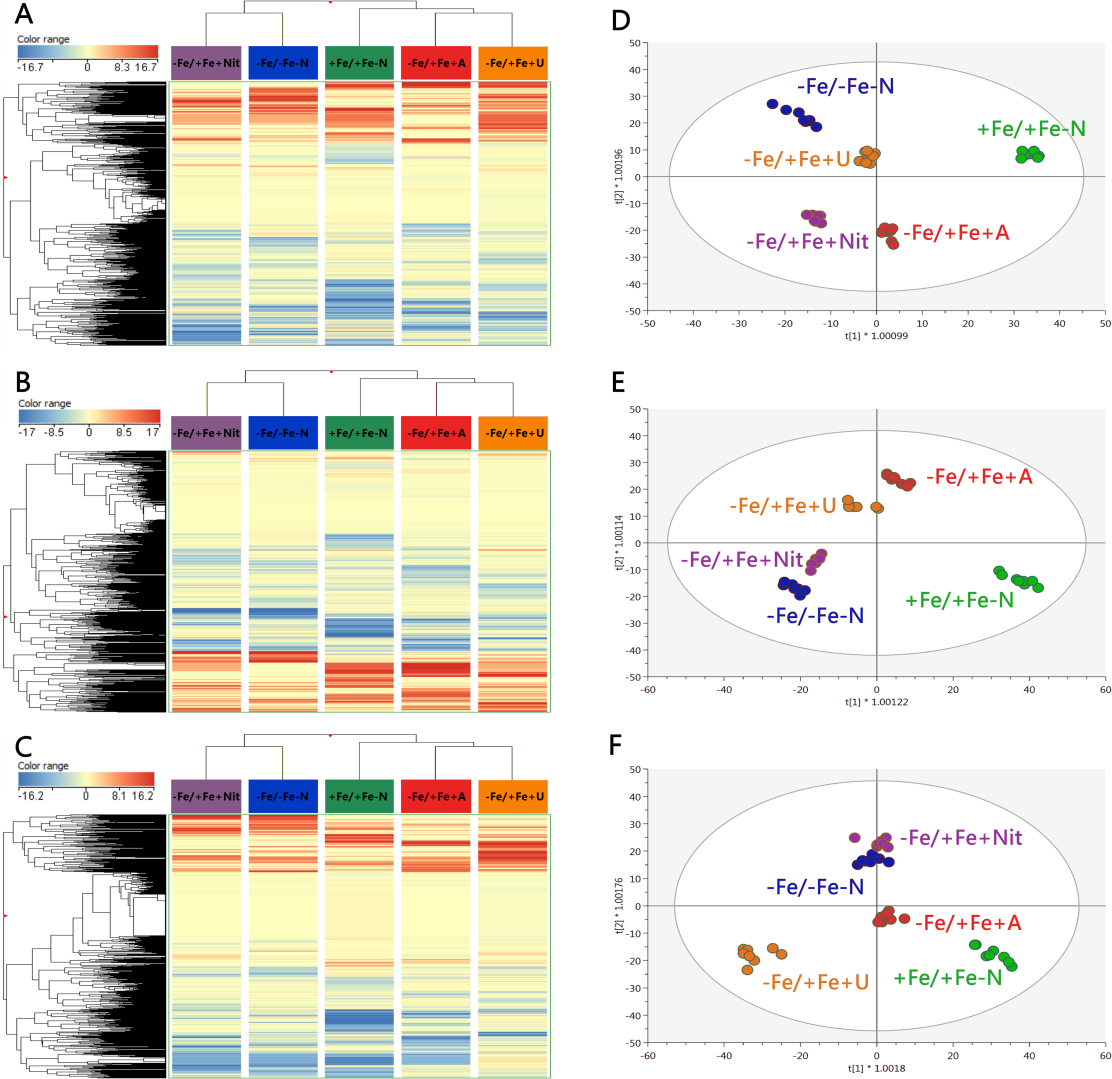
Unsupervised hierarchical cluster analysis carried out from ultra-performance liquid chromatography electrospray-ionization quadrupole time-of-flight mass spectrometry (UPLC ESI/QTOF-MS) metabolomic analysis of young **(A)** and old leaves **(B)** and roots **(C)** after N supply. The fold-change-based heat map was used to build hierarchical clusters (linkage rule: Ward; distance: Euclidean). Score plot of orthogonal projection to latent structure discriminant analysis (OPLS-DA) supervised modelling carried out on untargeted metabolomic profiles of young **(D)** and old leaves **(E)** and roots **(F)** after N supply. +Fe/+Fe-N, control with N deficiency; -Fe/-Fe-N, control with Fe and N deficiency; -Fe/+Fe+Nit, nitrate; -Fe/+Fe+A, ammonium; -Fe/+Fe+U, urea.

The application of untargeted metabolomics provided the annotation of 3320 chemical entities among the extracts derived from YL, OL, and roots of tomato plants. **Supplementary Table S4** shows the provides the list of annotated compounds in tomato samples, their abundance, molecular formula, composite mass spectra, and retention time. To decipher the influence of each factor involved in the metabolic profile of tomato plants, an unsupervised multivariate hierarchical cluster analysis (HCA) was first performed (**Supplementary Figure S5**). According to the similarity of metabolic profiles, the fold change-based heatmap showed that tissue played a clear role in clustering samples, grouping the profile associated with roots apart from that derived from leaves, suggesting a tissue-dependent response towards different N sources in tomato plants (**Supplementary Figure S5**). Consequently, further analyses were applied individually to each organ to provide insight into the impact of different N forms. Thus, the results from HCA of YL, OL, and roots are displayed in **Figure 2** (A, B, and C, respectively). In all cases, the same trend was observed for each tissue: according to their metabolic profile, Fe-deficient tomato plants treated with nitrate (-Fe/+Fe+Nit) showed a clear similarity with those cultured under the combined deficiency of nitrogen and iron (-Fe/-Fe-N), as they clustered together (**Figure 2**). Concerning the other subcluster, Fe-supplied plants (+Fe/+Fe-N) exhibited a distinctive profile, whereas those Fe-deficient plants treated with ammonium (-Fe/+Fe+A) and urea (-Fe/+Fe+U) showed a similar profile between them (**Figure 2**). These results suggest that 1) Fe deficiency shows a coordinated whole-plant impact; and 2) A and U supply may counter the effects of iron deficiency in tomato plants at a metabolic level.

Afterwards, to provide a distinctive perspective due to the application of different N sources under Fe deficiency, a supervised multivariate orthogonal projection to latent structures discriminant analysis (OPLS-DA) was performed for each tissue, and the obtained models for young and old leaves, and roots are shown in **Figure 2** (D, E, F, respectively). All models presented high-quality parameters in terms of goodness-of-fit (R^2^Y) and goodness-of-prediction (Q^2^Y): R^2^Y = 0.991 and Q^2^Y = 0.875 for the model of young leaves; R^2^Y = 0.985 and Q^2^Y = 0.865 for the model of old leaves; and R^2^Y = 0.979 and Q^2^Y = 0.894 for the model of roots. All models were also proved statistically significant at p < 0.001 (CV-ANOVA). Focusing on the discrimination between treatments, Fe-supplied plants exhibited an exclusive profile in all tissues as +Fe/+Fe-N was found apart from the rest of the treatments (**Figures 2D–F**). Considering the profiles of Fe-deficient plants, a high dependence on N sources was observed, following a tissue-dependent behavior. For young leaves, -Fe/+Fe+U promoted a similar profile to -Fe/-Fe-N, whereas -Fe/+Fe+Nit and -Fe/+Fe+A promoted distinctive profiles (**Figure 2D**). In the case of old leaves and roots, the profile from Nit-treated plants promoted a negligible difference with respect to those from -Fe/-Fe-N, whereas –Fe/+Fe+A drove the most differential profile compared to -Fe/-Fe-N in old leaves (**Figure 2E**). In comparison, -Fe/+Fe+U triggered the most distinctive profile in roots (**Figure 2F**).

The obtained OPLS models were combined with variable importance in projection (VIP) analysis to detect the metabolites with the highest discriminant power, the so-called VIP markers, which were determined by their VIP score. The full list of VIP markers is provided in **Supplementary Table S5**. In addition, a Venn diagram is provided in **Supplementary Figure S6** to graphically indicate the different and/or coincident VIP markers between tissues. The high number of metabolites exclusively associated with each tissue (a total of 78, 89 and 162 for young and old leaves and roots, respectively) confirms a clear tissue-dependent effect of N sources under Fe deficiency (**Supplementary Figure S6**). Moreover, YL and OL shared a total of 27 metabolites, suggesting a slightly similar modulation of their metabolic profiles, being mostly represented by stress-related metabolites, as shown for abscisic acid (ABA) derivatives and glucosinolates, as well as a wide range of metabolites closely related to N metabolism, i.e.: amino acids like Pro, and Ser and Glu derivatives, adenosine derivatives, and triferuloyl spermidine. Furthermore, both leaf tissues and roots also shared a series of discriminant N-containing compounds, represented by amino acid derivatives, such as histidinol and ornithine; nucleotide-derived metabolites, like those from adenine, uridine, cytidine, hypoxanthine and guanine; as well as some metabolites related to oxidative stress management, including glutathione derivatives and polyphenols, like daidzein, (-)-epicatechin, and cyanidin glycosides (**Supplementary Figure S6**).

**Figure 3:**
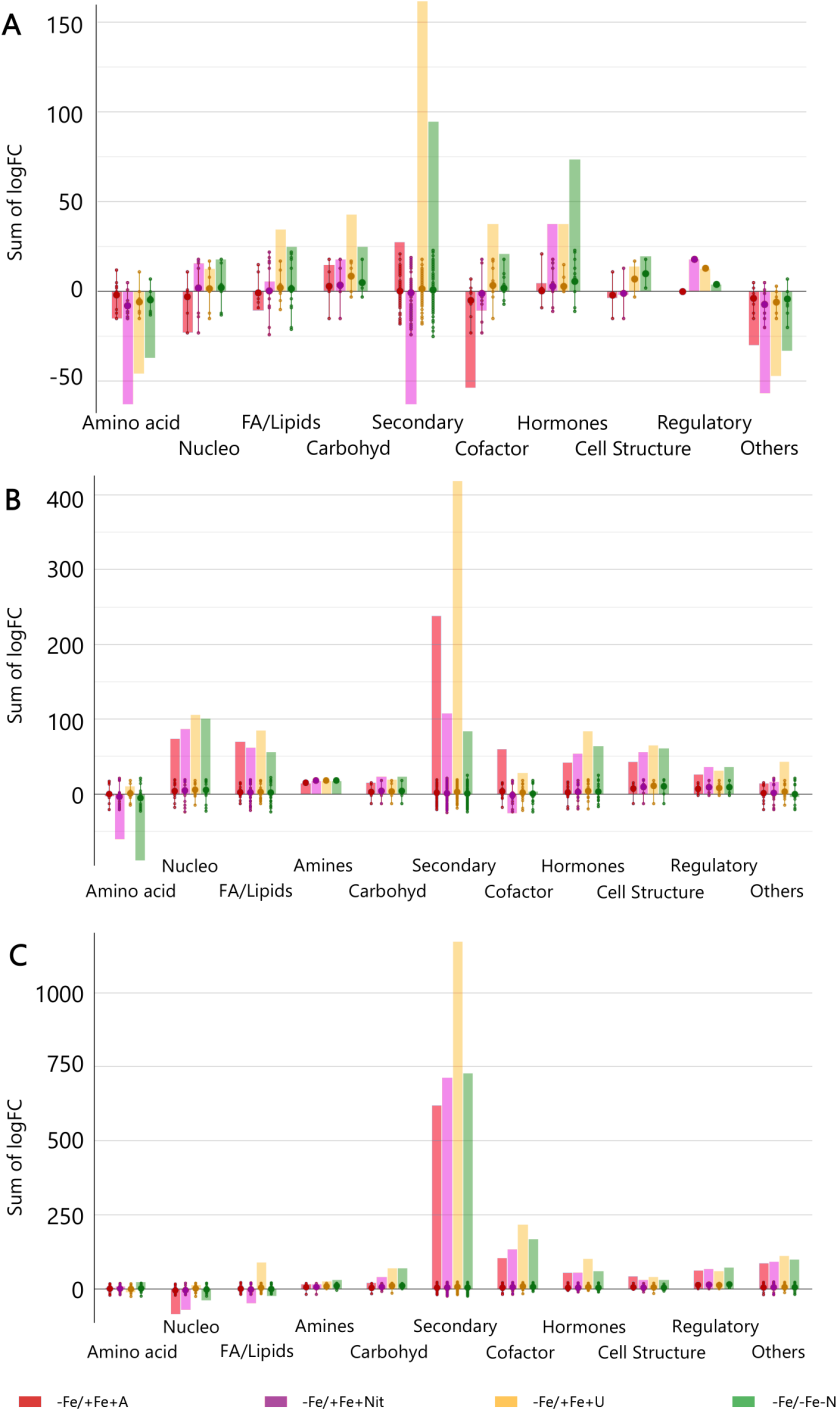
Biosynthetic pathways modulated by ammonium, nitrate, and urea in young leaves **(A)**, old leaves **(B)**, and roots **(C)** of Fe-deficient tomato plants. Significant metabolites (*p* < 0.01) and with fold-change (FC) values ≥ 2 for each treatment with respect to +Fe/+Fe-N were subjected to Pathway Analysis and visualized by the Omics Viewer Dashboard of the PlantCyc pathway Tool software (www.pmn.plantcyc.com). Large dots represent the average (mean) of all logFC for metabolites within the same subcategory, and the small dots represent the individual logFC values for each metabolite. The x-axis represents each set of subcategories. while the y-axis corresponds to the cumulative logFC. Amino acid, amino acids; Nucleo, nucleosides and nucleotides; FA/Lipids, fatty acids and lipids; Amines, amines and polyamines; Carbohyd, carbohydrates; Secondary, secondary metabolites; Cofactors, cofactors, prosthetic groups, electron carriers, and vitamins; Cell structure, cell structure-related metabolites; Regulatory, regulatory metabolites; Others: other metabolites. +Fe/+Fe-N, control with N deficiency; -Fe/-Fe-N, control with Fe and N deficiency; -Fe/+Fe+Nit, nitrate; -Fe/+Fe+A, ammonium; -Fe/+Fe+U, urea.

**Figure 4:**
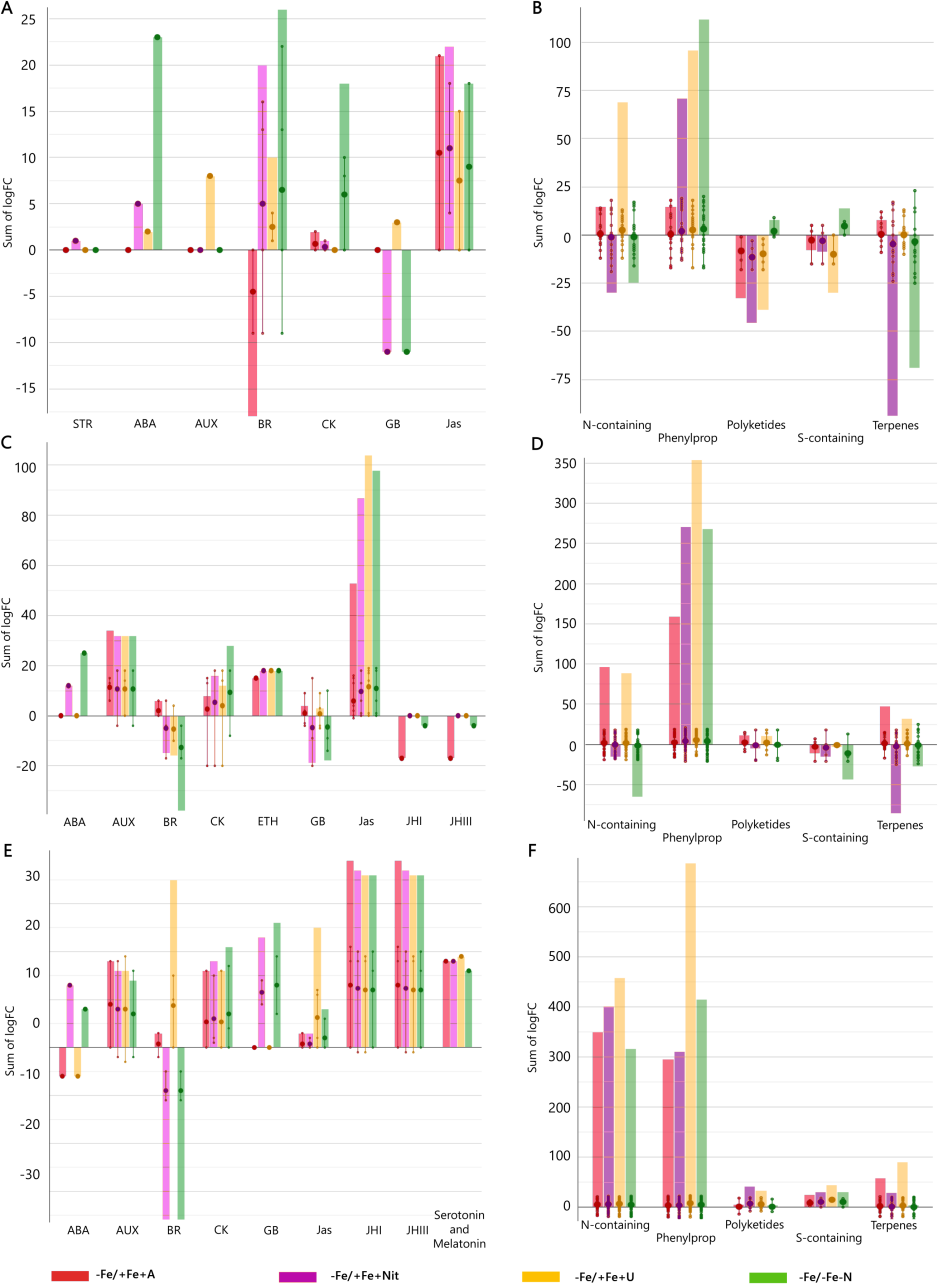
Hormone and secondary metabolite biosynthetic pathways modulated by ammonium, nitrate, and urea in young leaves **(A, B)**, old leaves **(C, D)**, and roots **(E, F)** of Fe-deficient tomato plants. Significant metabolites (*p* < 0.01) and with fold-change (FC) values ≥ 2 for each treatment with respect to +Fe/+Fe-N were subjected to Pathway Analysis and visualized by the Omics Viewer Dashboard of the PlantCyc pathway Tool software (www.pmn.plantcyc.com). Large dots represent the average (mean) of all logFC for metabolites within the same subcategory, and the small dots represent the individual logFC values for each metabolite. The x-axis represents each set of subcategories; while the y-axis corresponds to the cumulative logFC. STR, strigolactones; ABA, abscisic acid; AUX, auxins; BR, brassinosteroids; CK, cytokinins; GB, gibberellins and precursors; Jas, jasmonates; ETH, ethylene; JHI, juvenile hormone I; JHIII, juvenile hormone III; N-containing, N-containing secondary metabolites; Phenylprop, phenylpropanoids derivatives; S-containing, sulfur-containing secondary metabolites. +Fe/+Fe-N, control with N deficiency; -Fe/-Fe-N, control with Fe and N deficiency; -Fe/+Fe+Nit, nitrate; -Fe/+Fe+A, ammonium; -Fe/+Fe+U, urea.

Finally, to get insight into the effect of N source on the biosynthetic metabolic pathways of Fe-depleted tomato plants, the significant compounds (p < 0.01, FC ≥ 2) with respect to +Fe/+Fe-N (under N deficiency) were subjected to the PlantCyc Pathway Tools, and independently processed for young and old leaves and roots. **Figure 3** shows the modulation of biosynthetic metabolism for young and old leaves and roots of Fe-deficient tomato plants concerning Fe-supplied plants (+Fe/+Fe-N, **Figures 3A–C**, respectively). In general, Fe-deficient tomato plants exhibited an intense up-regulation of secondary metabolism, which was more evident in the case of urea supply, followed by a moderate induction of phytohormone biosynthesis (**Figure 3**). Given the importance of phytohormones and secondary metabolism in the response towards different N sources under Fe-deficient conditions, **Figure 4** includes the modulation of hormone biosynthesis and secondary metabolism in tomato plants.

Concerning young leaves, a general decrease in amino acid biosynthesis was observed, thus suggesting an impairment of N metabolism (**Figure 3A**). The induction of hormone biosynthesis under Fe deficiency in young leaves (**Figure 3A**) was mostly due to the increase of jasmonates and brassinosteroids biosynthesis (**Figure 4A**). In the case of jasmonates, all treatments strongly induced their biosynthesis, as observed for (-)-jasmonate (logFC = 15.88 – 21.86 for all treatments) and its precursor 3-oxo-2-(cis-2’-pentenyl)-cyclopentane-1-(3-oxooctanoyl)-CoA (**Figure 4A**). In the case of brassinosteroids, although A supply caused a decrease in their biosynthesis, the rest of the treatments (-Fe/-Fe-N > -Fe/+Fe+Nit > -Fe/+Fe+U) promoted their induction, being represented by brassinolide, 6-hydroxytaphasterol, and cathasterone derivatives (**Figure 4A**). Interestingly, young leaves from plants grown under the combined N and Fe deficiency (-Fe/-Fe-N) exhibited a sharp increase in abscisic acid accumulation (ABA, as shown for 2-trans-abscisate, logFC = 23.97) coupled with a decrease in gibberellins biosynthesis (gibberellin A_13_, GA_13_, logFC = -11.84), whose effects were similar in Nit-supplied plants (logFC = 5.39 for ABA and logFC = -11.84 for GA_13_). Regarding secondary metabolism (**Figure 4B**), all treatments under Fe-deficiency promoted the biosynthesis of phenylpropanoids, mostly represented by flavonoid and anthocyanin glycosides, following the trend (-Fe/-Fe-N > -Fe/+Fe+U > -Fe/+Fe+Nit > -Fe/+Fe+A). In contrast, the biosynthesis of N-containing compounds (NCCs) was found increased by the treatments -Fe/+Fe+U and -Fe/+Fe+A (average logFC = 2.56 and 0.56, respectively), which essentially involved alkaloids and glucosinolates, whereas a decrease was recorded by -Fe/+Fe+Nit and -Fe/-Fe-N (sum of logFC < -25.00 for both treatments; **Figure 4B**). Accordingly, -Fe/+Fe+Nit and -Fe/-Fe-N showed a similar effect by causing a pronounced decrease in terpenoid biosynthesis (average logFC = -4.70 and -3.45, respectively), thus reinforcing the parallel metabolic modulation driven by both treatments.

With respect to old leaves, the modulation of biosynthetic metabolism followed a similar trend to that observed in young leaves, exhibiting a clear induction of secondary metabolism and phytohormone biosynthesis by all treatments (**Figure 3B**). Regarding phytohormones, the biosynthesis of jasmonates was found increased by all treatments (**Figure 4C**), affecting a wide range of metabolites, such as 3-oxo-2-(cis-2’-pentenyl)-cyclopentane-1-(E-buta-2-enoyl)-CoA, 3-oxo-2-(cis-2’-pentenyl)-cyclopentane-1-(3R-hydroxybutanoyl)-CoA and related compounds (respectively: logFC = 4.27 and 4.29 for A, 13.77 and 13.94 for N, 17.92 and 18.14 for U, and 18.84 and 19.15 for -Fe/-Fe-N). In parallel, the biosynthesis of the cytokinin isopentenyladenine-7-N-glucoside was found to be strongly decreased by all treatments (logFC = -20.28 – -8.48; **Figure 4C**). Again, as observed for young leaves, Nit-supplied and -Fe/-Fe-N plants showed a similar influence on the phytohormonal profile of tomato plants under Fe deficiency (**Figure 4C**), since both drove the induction of 2-trans-abscisate biosynthesis (logFC = 12.41 and 25.44, respectively) and the repression of brassinosteroids (such as 3-epi-6-deoxycathasterone, logFC = -17.09 for both treatments) and gibberellins biosynthesis, as reported by gibberellins A_3_ (logFC = -9.08 and -9.07, respectively) and A_36_ (logFC = -20.06 and -14.86, respectively). Focusing on secondary metabolism, the same results were observed with respect to young leaves (**Figure 4D**) since all Fe-deficient plants exhibited an increase in phenylpropanoid biosynthesis, essentially represented by flavonoid glycosides and stilbenes. Furthermore, the biosynthesis of NCCs and terpenes was increased by -Fe/+Fe+A and -Fe/+Fe+U, whereas -Fe/-Fe-N and -Fe/+Fe+Nit treatments provoked the repression of both pathways.

Considering roots, the impact of Fe deficiency again caused significant induction of secondary metabolism and phytohormone biosynthesis, as reported for leaves (**Figure 3C**). In the case of phytohormones, all Fe-deficient treatments increased cytokinins biosynthesis, especially kinetin-7-*N*-glucoside, which exhibited logFC = 15.23 – 17.18 in all treatments (**Figure 4E**). Again, a parallel behavior was reported to roots from Nit-supplied plants and those grown under Fe and nitrogen combined deficiency (-Fe/-Fe-N). Both treatments, -Fe/+Fe+Nit and -Fe/-Fe-N, elicited the biosynthesis of 2-trans-abscisate (logFC = 13.78 and 8.87, respectively), gibberellins A_36_ (logFC = 14.58 and 19.89, respectively) and A_37_ (logFC = 9.64 and 7.25, respectively), and the jasmonate precursor 3-oxo-2-(cis-2’-pentenyl)-cyclopentane-1-(3-oxooctanoyl)-CoA (logFC = 2.31 and 6.76, respectively), whereas they strongly inhibited brassinosteroids biosynthesis, mostly represented by campesterol derivatives, such as (6α)-hydroxycampestanol and campest-5-en-3-one (logFC = -11.93 and -5.25 for both treatments for each compound, respectively). U and A supply countered those effects since these treatments (-Fe/+Fe+U and -Fe/+Fe+A) promoted a decrease in the biosynthesis of 2-trans-abscisate (logFC < -6.22 for both treatments), meanwhile they boosted the biosynthesis of brassinosteroids, especially in U-supplied roots, logFC = 10.79, 15.14, and 10.79 for (6α)-hydroxycampestanol, campest-5-en-3-one, and 3-epi-6-oxocathasterone, respectively (**Figure 4F**). Considering secondary metabolism, roots from Fe-deficient tomato plants showed a similar effect among all treatments, boosting the biosynthesis of phenylpropanoids, mainly flavonoid glycosides, and NCCs, represented by alkaloids and glucosinolates and their derivatives, where U-treated roots exhibited the highest cumulative logFC values (**Figure 4F**).

Overall, Fe deprivation promoted a general induction of plant stress, reported by the strong biosynthetic induction of stress-related phytohormones, such as jasmonates and abscisic acid derivatives, as well as important secondary metabolites implicated in plant stress management, as is the case of phenylpropanoids, glucosinolates, and alkaloids. In leaves, the stress associated with Fe-deficiency was partially reverted by U and A as N sources, whereas Nit supply play a negligible effect, showing an impairment of phytohormone biosynthesis and nitrogen metabolism comparable to that of Fe and N-deficient plants. The same behavior could be attributed to roots, where Nit supply promoted the accumulation of stress-derived phytohormones, such as ABA and jasmonates derivatives in Fe-deficient tomato plants. Such a stress-inducing fingerprint was also countered by the supply of both U and A, which also boosted the biosynthesis of brassinosteroids, phenylpropanoids, and NCCs compared to Fe-supplied, nitrogen-deficient tomato plants.

### Ferric-chelate reductase activity

**Figure 5:**
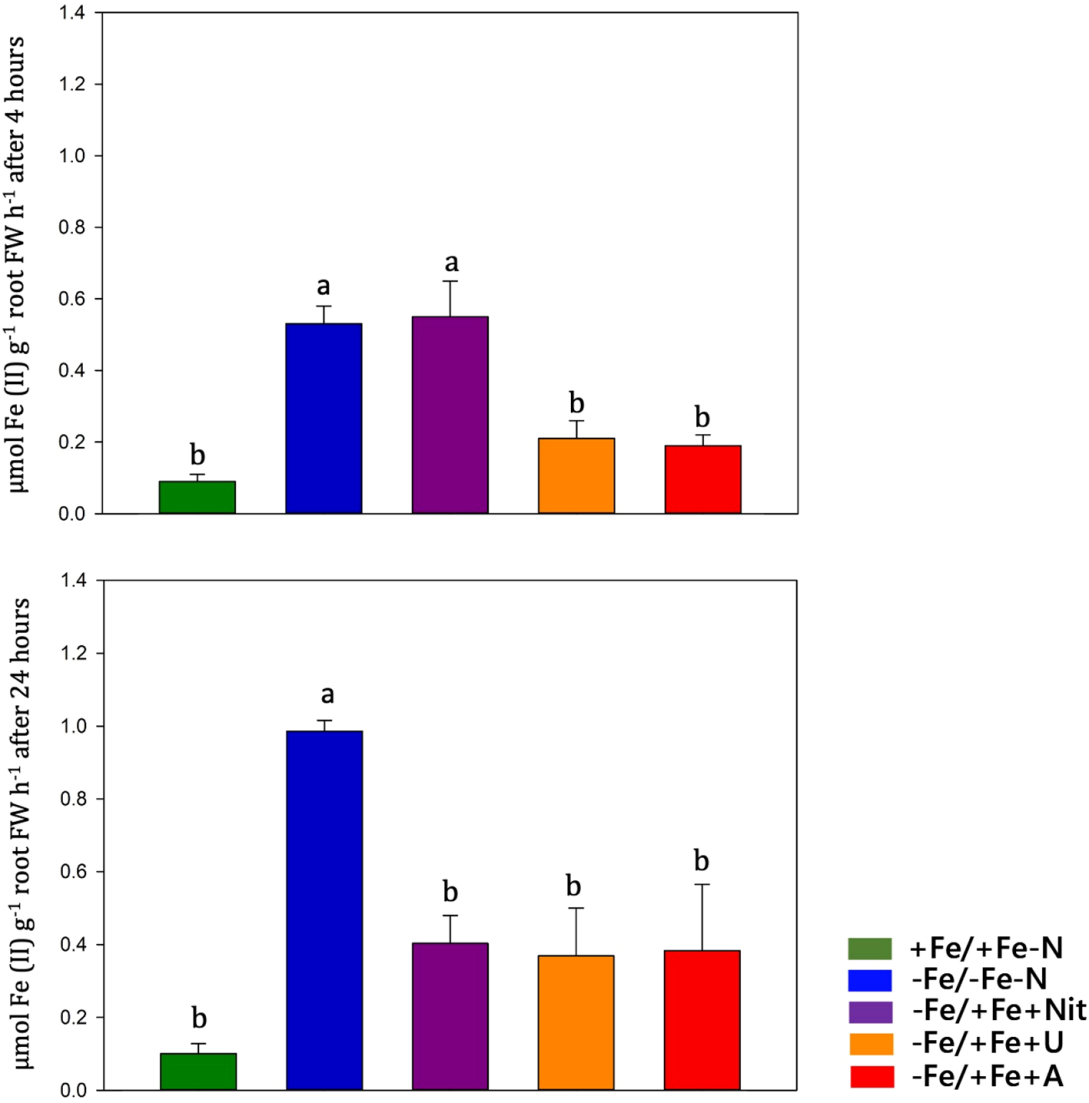
Ferric chelate reductase (FCR) activity quantification (expressed in µmol Fe (II) g^-1^ root FW h^-1^) in tomato roots measured at 4 and 24 hours after treatment (+Fe/+Fe-N, green; -Fe/+Fe-N, blue; -Fe/+Fe+Nit, purple; -Fe/+Fe+U, orange; -Fe/+Fe+A, red).

Fe(III)-chelate reductase activity was measured on intact tomato roots at 4 and 24 hours after treatment (**Figure 5**). Under Fe-sufficient condition (+Fe/+Fe-N), tomato roots displayed low FCR activity values, whereas high FCR activity values were observed under Fe deficiency. Under Fe resupply, Fe deficient plants (-Fe/-Fe-N) operated a feedback regulation on previously activated mechanisms, such as FCR activity. The timing of these feedback regulations was dependent on the N form applied: under urea (-Fe/+Fe+U) and ammonium (-Fe/+Fe+A), the FCR activity was slowed down already after 4 hours from the Fe-resupply, whereas under nitrate (-Fe/+Fe+Nit) the FCR activity was reduced after 24 hours.

### Gene expression analyses

**Figure 6:**
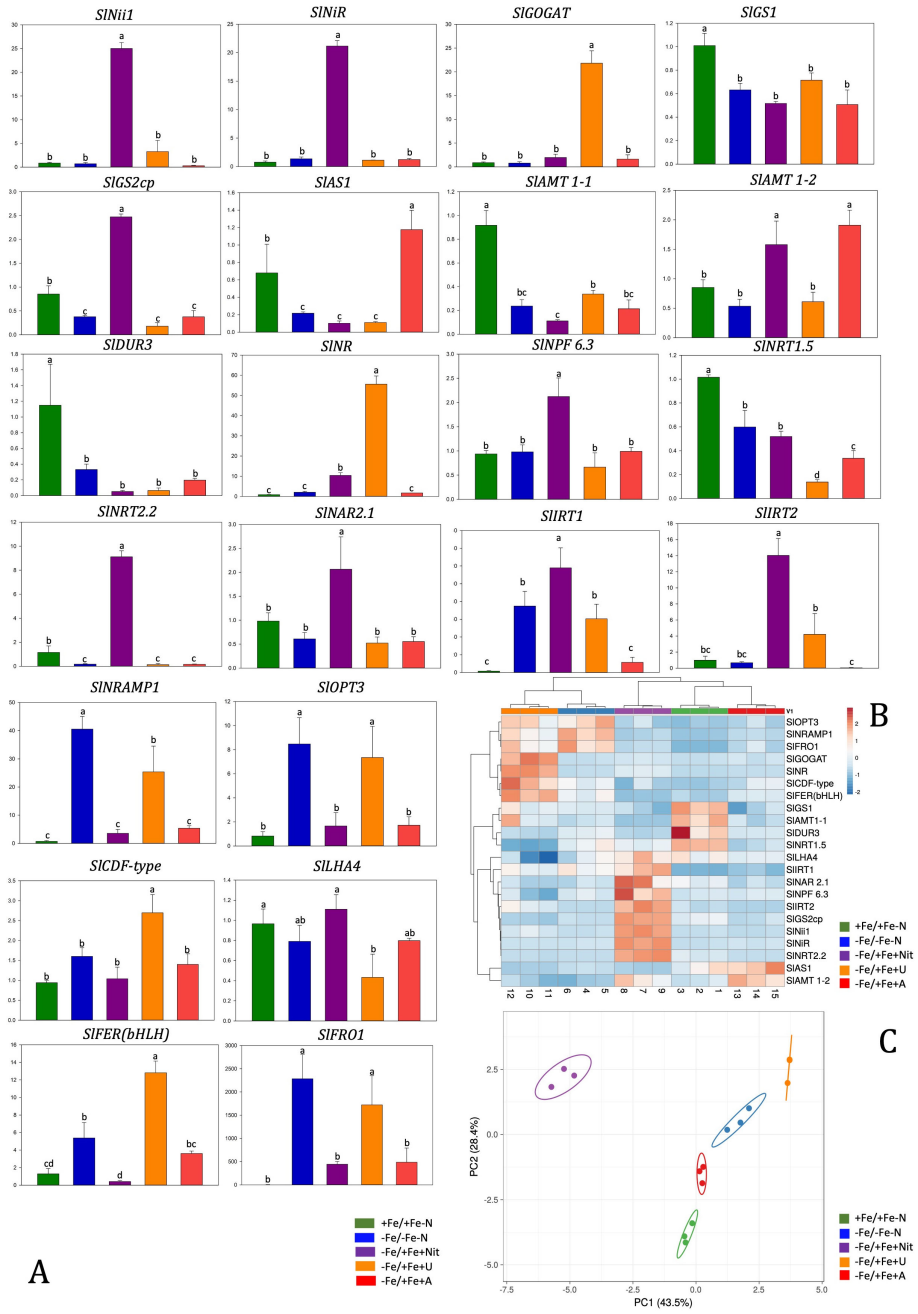
Relative gene expression level of tomato roots after 24 hours of treatment with different N sources and with Fe resupply **(A)**. Data were referred to the averaged expression of two housekeeping genes *SlEF1* and *SlUbi.* Relative changes in gene transcript levels were referred to the average transcript level of housekeeping genes in +Fe/+Fe-N roots (relative gene expression = 1). Letters refer to statistical significance (Holm-Sidak ANOVA, N= 3, p-value< 0.05). In the heatmap, a clustering of relative gene expression and samples in tomato roots **(B)**. PCA analyses show principal component 1 and principal component 2 that explain: 43.5% and 28.4% of the total variance in root **(C)**.

Gene expression analysis was performed at 24 hours after treatments on tomato roots by real-time RT-PCR and showed differences among treatments in the expression of key genes involved in Fe and N acquisition and utilization that can be well visualized by PCA (PERMANOVA p<0.001) and by heatmap clustering analysis (**Figures 6A–C**). These analyses were performed on twenty-two genes coding for: Fe and heavy metal transporters (*SlIRT1, SlIRT2, SlNRMAP1, SlCDF-type)*, proteins involved in Fe assimilation (*SlLHA4, SlOPT3*), a ferric reductase oxidase (*SlFRO1*), nitrate transporters with one of their accessory proteins (*SlNRT2.2, SlNRT1.5, NPF6.3, SlNAR2.1*), ammonium transporters (*SlAMT1-1, SL AMT1-2)*, urea transporter (*SlDUR3*) and N assimilatory enzymes (*SlNii1, SlNiR, SlGS2, SlGS1, SlNR, SlGOGAT, SlAS*).

Nitrate (-Fe/+Fe+Nit) treatment led to an increase of relative gene expression level of *SlIRT1, SlIRT2, SlNii1,SlNPF6.3, SlNiR, SlGS2cp, SlNRT2.2* in comparison to other N-treatment and controls (+Fe/+Fe-N, -Fe/-Fe-N), while was observed a downregulation of *SlNRAMP1, SlOPT3, SlLFER(bHLH), SlFRO1* if compared with -Fe/-Fe-N and downregulation of *SlGS1, SlAS1, SlAMT1-1, SlDUR3, SlNRT1.5* if compared with +Fe/+Fe-N.

Regarding the effect of ammonium treatment (-Fe/+Fe+A), the genes *SlAS1 and AMT 1-2* were significantly upregulated compared to the controls (+Fe/+Fe-N, -Fe/-Fe-N). On the other hand, the supply of A led to a downregulation of *SlGS1, SlGS2cp* and *SlNRT1.5* genes compared to +Fe/+Fe-N.

Urea treatment (-Fe/+Fe+U) resulted in a significant upregulation of *SlGOGAT, SlNR, SlCDF-type* and *SlFER(bHLH)* in comparison to other N-treatment, +Fe/+Fe-N and -Fe/-Fe-N. Moreover, a similar pattern to -Fe/-Fe-N was observed concerning *SlNRMAP1, SlOPT3* and *SlFRO1* genes that resulted in being upregulated in -Fe/-Fe-N and -Fe/+Fe+U treatment in comparison to +Fe/+Fe-N, -Fe/+Fe+Nit or -Fe/+Fe+A.

## Discussion

### Iron deficiency response

Before starting the treatment of N and Fe resupply, 42-day-old plants displayed visible symptoms and molecular evidence of Fe shortage and N limitation in agreement with literature (Zamboni et al., 2012; Sainju et al., 2003; Zamboni et al., 2016).

After 24 hours, the Fe resupplied plants (-Fe/+Fe+A, -Fe/+Fe+U, -Fe/+Fe+Nit) displayed an increase in Fe content and a decreased FCR activity compared to the control plants (-Fe/-Fe-N). These results suggest that Fe-deficient plants could use the resupplied Fe and indicate the occurrence of a feedback regulation of Fe responsive genes by Fe resupplied along with nitrate or ammonium.

In roots, nitrate and ammonium treatments exhibited similar gene expression patterns except for *IRT1* and *IRT2*, which were found both upregulated by nitrate compared to the other treatments. The upregulation of *IRTs* by nitrate agrees with evidence from the literature (Liu et al., 2015) and explains the high concentration of Fe measured in plants. In contrast to the reduced N forms (urea or ammonium), nitrate seems to delay the activation of the retro regulation on FCR activity (which was still high after 4 hours). This longer activation maybe due to the higher pH of the apoplast in nitrate-fed plants therefore to an inhibition of FCR activity (Nikolic and Römheld, 2003). Regarding urea, even after 24 hours of Fe resupply, root maintained upregulated several genes involved in *Strategy I*, such as *FRO1, IRT1, NRAMP1, OPT3*, and *FER*. In *Arabidopsis*, Mérigout et al. (2008) observed that *FRO* and *IRT* genes were positively regulated by urea treatment compared to the inorganic N forms (ammonium, nitrate). Thus, urea might have a *per se* effect at the transcriptional level on some components of *Strategy I*. Considering the high Fe concentration in roots, this transcriptional pattern suggests that the urea acquisition pathway interacts with the one of Fe, promoting the acquisition of the micronutrient and its use by plants. The unalike response of *SlFRO1* expression and FCR activity could be ascribable to the contribution of other *SlFRO* isoforms to the enzymatic activity or to a post-transcriptional modulation (Connolly et al., 2003; Jeong and Connolly, 2009). It is interesting how FER is upregulated by urea and Fe supply, as its expression level was even higher than those recorded in -Fe/-Fe-N (Fe-deficient plants). This transcription factor plays a key role in activating the Fe-deficiency response by inducing the expression of genes involved in the Fe-uptake system (i.e. *FRO* and *IRT*; Ling et al., 2002; Brumbarova and Bauer, 2005). Thus, its upregulation only by urea (and not by other N forms) might explain the absence of feedback regulation on *Strategy I* components after 24 hours from the Fe resupply. Based on these observations, we can suppose that the effect of the three N forms depends on the gene expression, orchestration and timing of the feedback regulation on the Strategy I components.

### The form of N also affects the accumulation of other nutrients

Being Fe an essential co-factor for N assimilation is plausible to state that Fe-nutritional status influences N uptake and content in plants. Parveen et al. (2018) observed that the Fe supply improved the N uptake and accumulation in roots. Under our experimental conditions, a higher N content was measured in nitrate and ammonium Fe-resupplied roots (-Fe/+Fe+Nit, -Fe/+Fe+A), suggesting a different acquisition rate linked to the N-form supplied and/or a different promptness of reaction to Fe presence in the nutrient solution.

Significant interactions between S and Fe homeostasis have been widely studied and described in several crops, together with the one between S and N, in both grasses and dicots (Varin et al., 2010; Ciaffi et al., 2013; Paolacci et al., 2014; Wu et al., 2015; Zuchi et al., 2015; Coppa et al., 2018). Ammonium induced a higher S concentration in leaves than other N treatments in our conditions. This behavior might result from a competition effect between nitrate and sulphate anions for reducing equivalents needed for their assimilation (De Bona et al., 2011; Kruse et al., 2007).

Under Fe deficiency, plants display changes in the composition of other micronutrients, such as Cu, Mn and Zn (Rai et al., 2021), probably due to the capability of FRO and IRT to mediates also the acquisition of other metals (Korshunova et al., 1999; Connolly et al., 2002). Indeed, several studies reported that these metals influence each other’s fate inside the plant, and they also compete for metal transporters’ a specific activities, such as IRT1 and NRAMPs (Bashir et al., 2016; Grotz and Guerinot, 2006; Rai et al., 2021).

Regarding Cu, its concentration increased under Fe deficiency in comparison to Fe sufficient ones in all organs (roots, OL, YL). In leaves this response was previously observed in several plant species both grasses and dicots (Waters and Troupe, 2012; Waters et al., 2006; Welch et al., 1993; Valdés-López et al., 2010; Suzuki et al., 2006; Chaignon et al., 2002). It has been reported that a high level of Fe availability reduces the acquisition of Zn while Fe-shortage determines Zn, Mn and Cu accumulation due to the induction of bivalent metal transporters, such as *SlIRT1* and *SlNRAMP1* (Arrivault et al., 2006; Eckhardt et al., 2001; Waters and Armbrust, 2013; Ray et al., 2014; Saenchai et al., 2016; Vert et al., 2002; Zhang et al., 1991). In agreement with the cited literature, overall, all Fe-deficient plants showed higher Zn concentration in leaves (as well as Mn) in comparison to +Fe/+Fe-N, especially Nit-treated plants (-Fe/+Fe+Nit). Previous work performed on tomato plants grown under Fe sufficient conditions highlighted that the application of urea led to high concentrations of these micronutrients in plants (Lodovici et al., 2024). These findings suggest that an interplay between N-form and Fe-availability concurs to shape these nutrient profiles.

### N forms interact with the primary metabolism in the Fe resupply response

Regarding the effect of Fe nutritional status on N acquisition in plants, only fragmented information is available, especially referring to the changes in N metabolism that occurs under Fe-deficiency conditions. Rellán-Álvarez et al. (2011) stated that the main changes in the metabolite profile of Fe-deficient leaves include a consistent increase in amino acid (AA) and N-related metabolite content. In agreement with literature (Holley and Cain, 1955), the Fe deficiency response determined an overall increase of some amino acid concentrations in comparison to Fe-sufficient plants depending on the available N-form. In particular, results indicate an increase of glutamine- and glutamate-related compounds in roots. The high concentration of glutamine in urea or ammonium treated roots suggests a fast assimilation of N in this organ that could occur by a cytosolic N assimilatory pathway (cytosolic GS1 and ASN, Buoso et al., 2021a, b) rather than by the plastidial one.

In agreement with our results (arginine);, Another amino acid is known to be responsive to Fe deficiency is arginine (Holley and Cain, 1955), which was found more concentrated in -Fe/-Fe-N, -Fe/+Fe+Nit and -Fe/+Fe+A and less in -Fe/+Fe+U. Interestingly, the arginine concentration under Fe sufficiency condition was found to be responsive only to urea occurrence in nutrient solution and not to the presence of other inorganic N forms (Lodovici et al., 2024). The higher concentration of arginine can be related to the catabolic processes and in particular to the arginine cycle that mediates the degradation of arginine to produce L-ornithine and urea (Girard-Thernier et al., 2015). This hydrolytic reaction is mediated by arginase, which activity is dependent to divalent cation as cofactor (ferrous ion in yeast arginase, Middelhoven et al., 1969). We can speculate that the higher arginine concentration in Fe-deficient plants are the consequence of a reduction in the arginase activity, and this effect is less present in urea-treated plants due to a redistribution of the metal with a positive effect on arginase activity and maybe a inhibition of the arginase due to the accumulation of its product, urea.

### Interplay between Fe and N on secondary metabolism phytohormones

It is well known that phytohormones have a role in plant stress responses to both biotic and abiotic stresses (Checker et al., 2018; Divte et al., 2021; Banerjee and Roychoudhury, 2022). In general, the plant response to Fe-limiting conditions are positively regulated by auxins, ethylene, gibberellins, and nitric oxide and negatively controlled by cytokinins, abscisic acid, brassinosteroids (BRs) and jasmonic acid (Rai et al., 2021). Auxin and ethylene are involved in root hair proliferation (Hindt and Guerinot, 2012) and in the control of root growth by nitric oxide and auxins (Ramírez et al., 2008). Wang et al. (2012b) reported that BRs are involved in inhibiting Fe uptake as it can be observed in -Fe/+Fe+A plants showing a lower Fe concentration in roots in comparison to other N forms. In fact, the application of BRs to cucumber seedlings resulted in a substantial limited increase in FRO activity under Fe deficiency (Wang et al., 2012b). A different modulation in the BRs biosynthetic pathway has been observed across treatments (Nit, U, A) and tissues confirming their role in the Fe-deficient response. In Fe-sufficient condition all three N-forms led to the same modulation on that biosynthetic pathway (Lodovici et al., 2024) suggesting that Fe availability influences how N forms affect the biosynthesis of BRs in plants. On the other hand, gibberellins (GA) positively regulate Fe uptake by promoting the induction of *FRO2* and *IRT1* in *Arabidopsis* (Matsuoka et al., 2014). Moreover, ethylene and nitric oxide positively induce the expression of *IRT1* and *FRO2*, suggesting that these two signals increase the sensitivity of plants towards Fe uptake (García et al., 2010; Graziano and Lamattina, 2007; Lucena et al., 2006; Waters et al., 2007). Cytokinins led to a down-regulation of the two genes (Séguéla et al., 2008). Hormonal influence on Fe acquisition gene expression may serve to coordinate physiology and stress responses with necessary adaptations for altered root growth and Fe uptake (Schikora and Schmidt, 2001; Schmidt, 1999; Schmidt et al., 2000). In our experimental conditions, changes in the accumulation of phytohormones and phytohormone-related compounds were observed suggesting a different timing of plant responses to Fe-deficiency conditions.

Both biotic and abiotic stresses, such as nutritional stresses, often lead to phenylpropanoid accumulation (Mai and Bauer, 2016). According to the literature, plants under Fe-deficiency promoted the biosynthesis of phenylpropanoids (e.g. lignin and suberin precursors, flavonoids and anthocyanin glycosides) in all the analyzed plant organs, especially those treated with urea (-Fe/+Fe+U) that showed the highest accumulation, especially in roots followed by A (-Fe/+Fe+A), Nit (-Fe/+Fe+Nit) and then -Fe/-Fe-N.

## Conclusions

The obtained results coupled with the information available in the literature, suggesting different promptness and regulation of tomato plants adaptation mechanisms to the Fe-deficiency conditions strictly related to a specific plant organ and to the applied N-forms. During the Fe supply, the N forms alter differently the primary metabolism (particularly amino acids), secondary metabolisms, and hormones, leading to changes in the morphology, physiology, and exudation. These processes are relevant to define rhizosphere conditions and hence they contribute to define the Fe bioavailability for the root uptake.

## Data Availability

The original contributions presented in the study are included in the article/**Supplementary Material**. Further inquiries can be directed to the corresponding author.

## Supplementary material

Supplementary Table 4 Dataset from untargeted metabolomics of leaves and roots after nitrogen supply. Compounds are presented with individual intensities, molecular formulas, retention time, and composite mass spectra.

Supplementary Table 5 Discriminant metabolites identified by the VIP analysis in leaves and roots after nitrogen supply. Compounds were selected as discriminant by possessing a VIP score > 1.3.

Supplementary Table 6 VIP markers associated with Venn diagram regions from leaves and roots after nitrogen supply.

Supplementary Table 7 Significant compounds (*p-value* < 0.01, FC ≥ 2) with respect to +Fe/+Fe-N (under nitrogen deficiency) subjected to the Plantcyc Pathway Tools for young and old leaves and roots from Fe-deficient tomato plants.
