# Supplementary material for: A multi-omics insight on the interplay between iron deficiency and N forms in tomato
Source: Front Plant Sci. 2024 Oct 16;15:1408141. doi: 10.3389/fpls.2024.1408141 (PMC11521840; doi:10.3389/fpls.2024.1408141)
Supplement: Supplementary file 1 [file DataSheet1.pdf]

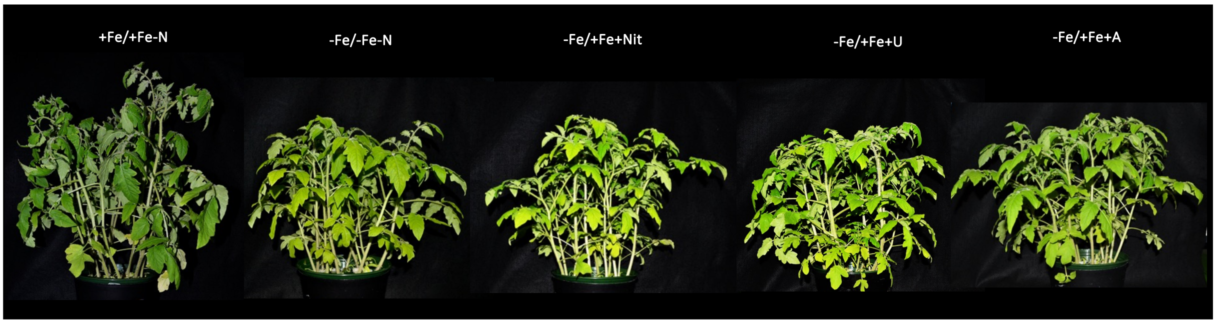

Supplementary Figure S1. Morphological representation of 43-day-old tomato plants treated with different N-sources and Fe-resupply.

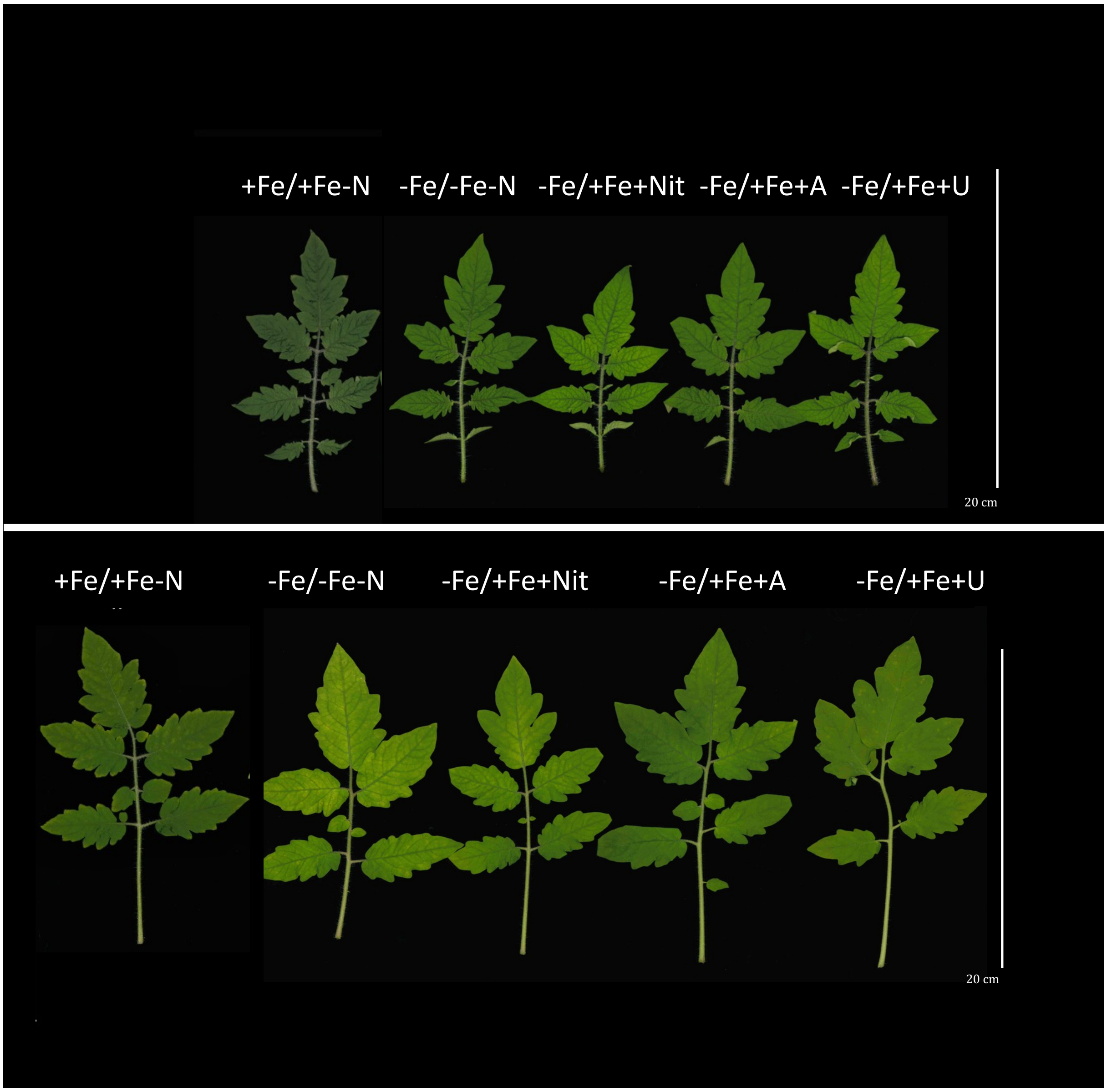

Supplementary Figure S2. Morphological representation of Young Leaves and Old Leaves per each nitrogen and Fe-resupplied treatment (Fe-sufficient control (+Fe/+Fe-N), Fe-deficient control (-Fe/-Fe-N), nitrate (-Fe/+Fe+Nit), urea (-Fe/+Fe+U), ammonium (-Fe/+Fe+A)).

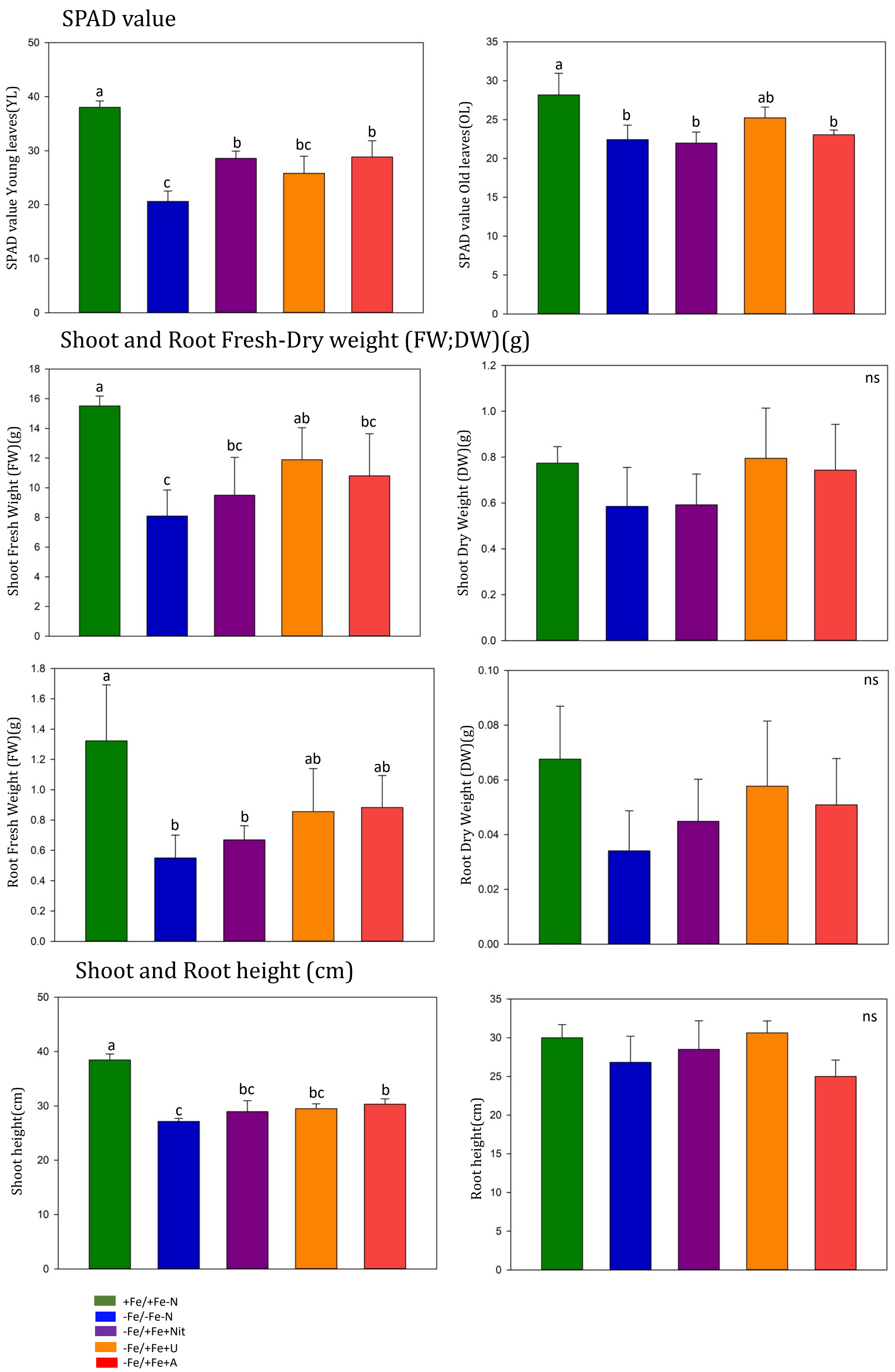

Supplementary Figure S3. SPAD value related to tomato plants grown in different N and Fe conditions (Fe-sufficient control (+Fe/+Fe-N), Fe-deficient control (-Fe/-Fe-N), nitrate (-

Fe/+Fe+Nit), urea (-Fe/+Fe+U), ammonium (-Fe/+Fe+A)) and referred to young (YL) and old (OL) leaves, respectively. Shoot and root height (cm) and Fresh and dry weights of 43-day-old shoots and roots. In the histograms, letters refer to statistical significance differences (Holm-Sidak ANOVA, N= 3, p-value< 0.05).

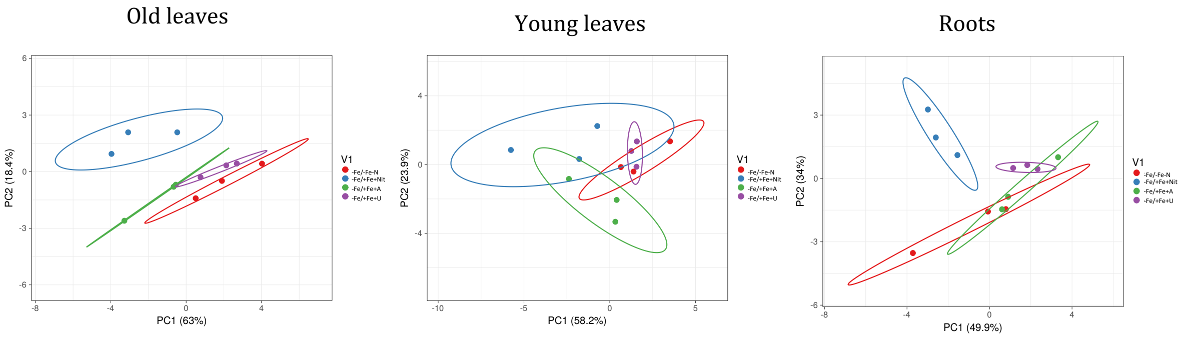

Supplementary Figure S4. PCA analyses, performed on nutritional element content of Fe-deficient/resupplied plants (-Fe/-Fe-N, -Fe/+Fe+Nit, -Fe/+Fe+U, -Fe/+Fe+A), show principal component 1 and principal component 2 that explain: 63% and 18,4% of the total variance in old leaves (A), 58,2% and 23,4% of the total variance in young leaves (B) and 49,9% and 34% of the total variance in roots (C).

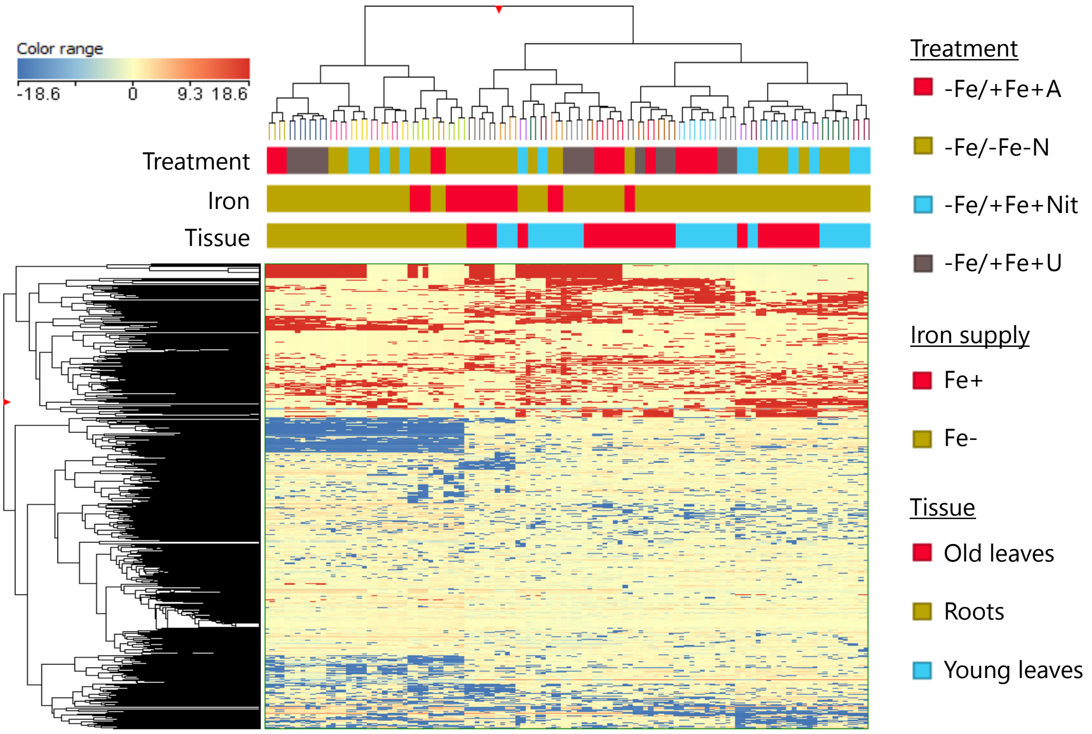

Supplementary Figure S5. Unsupervised hierarchical cluster analysis carried out by ultra-performance liquid chromatography electrospray ionization quadrupole time-of-flight mass spectrometry (UHPLC-ESI/QTOF-MS) metabolomics analysis of tomato samples after nitrogen and Fe-resupply. The fold-change-based heat map was used to build hierarchical clusters (linkage rule: Ward; distance: Euclidean).

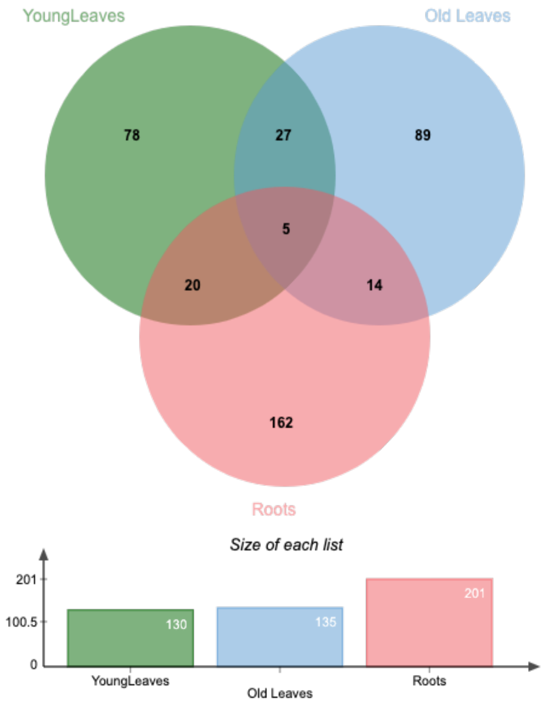

Supplementary Figure S6. Venn diagram showing the distribution of discriminant VIP markers between old and young leaves and roots of tomato plants after N supply. The corresponding list of compounds for each region is provided in Supplementary Table S4.
