# Supplementary material for: A multi-omics insight on the interplay between iron deficiency and N forms in tomato
Source: Front Plant Sci. 2024 Oct 16;15:1408141. doi: 10.3389/fpls.2024.1408141 (PMC11521840; doi:10.3389/fpls.2024.1408141)
Supplement: Supplementary file 2 [file Table1.docx]

**Supplementary Table S1.** Experimental plan. In the table, the name of treatments and the nutritional conditions applied for 1 week (w) to 35-day-old plants and during the 24 hours of treatment are shown. Before the 24 hours of treatments all plants were exposed to 1 week of N deficiency. Before the 24 hours of treatment, +Fe/+Fe-N plants refer to Fe-sufficient and N-deficient plants; -Fe/-Fe-N, -Fe/+Fe+Nit, -Fe/+Fe+U, -Fe/+Fe+A plants refer to Fe-deficient and N-deficient plants. For a comprehensive description of the treatments, please refer to the Materials and Methods section.

| **Treatment ID** | **35-42 days old (1 w)** | **24 hours of treatment** | |
| --- | --- | --- | --- |
|  |  | **N** | **Fe** |
| **+Fe/+Fe-N** | 0 mM N, 0.1 mM Fe-EDTA | 0 mM N | 0.1 mM Fe-EDTA |
| **-Fe/-Fe-N** | 0 mM N, 0 mM Fe | 0 mM N | 0 mM Fe |
| **-Fe/+Fe+Nit** | 0 mM N, 0 mM Fe | 2 mM nitrate | 0.005 mM Fe-EDTA |
| **-Fe/+Fe+U** | 0 mM N, 0 mM Fe | 2 mM urea | 0.005 mM Fe-EDTA |
| **-Fe/+Fe+A** | 0 mM N, 0 mM Fe | 2 mM ammonium | 0.005 mM Fe-EDTA |
