# Supplementary material for: A multi-omics insight on the interplay between iron deficiency and N forms in tomato
Source: Front Plant Sci. 2024 Oct 16;15:1408141. doi: 10.3389/fpls.2024.1408141 (PMC11521840; doi:10.3389/fpls.2024.1408141)
Supplement: Supplementary file 3 [file Table2.docx]

**Supplementary Table S2.** Lists of primers used for Real-time RT-PCR analysis

| **Gene** | **NCBI Reference Sequence** | **Name** | **Forward primer 5’-3’** | **Reverse primer 5’-3’** |
| --- | --- | --- | --- | --- |
| *SlAS1* | NM_001319849.2 | Asparagine synthetase 1 (AS1) | GATCCTTCTGGTAGGGCTGC | GCCTACCATCCTTGGCATGT |
| *SlAMT1-2* | NM_001247324.2 | Ammonium transporter 1-2 (AMT1-2) | TGGATTTATCGCAGCTTGGGT | TATCCCCCATGAACCGCAAC |
| *SlGS1* | NM_001319855.1 | Glutamine synthetase 1  (GS1) | GACAGAAGGCCAGCCTCAAA | AAGCGATCCCTCGAGCAAAT |
| *SlAMT1-1* | NM_001317738.1 | Ammonium transporter 1-1 (AMT1-1) | GGGTTCGTAGCGGCTTTAGT | ATCCCCCATGCACCACAAC |
| *SlDUR3* | XM_004245951.4 | Urea-proton symporter (DUR3) | CAACAGTGCCAAAGCATGGA | TGCTAAATTGCCCCACAGGA |
| *SlNRT1.5* | XM_004244498.4 | Nitrate transporter 1.5  (NRT 1.5) | CGGATGGGTATTGGACTGGT | GAGCCTTCACAGTGTTTGCA |
| *SlNPF6.3* | XM_004245698.4 | Nitrate trasnporter 1.1  (NRT1.1) | ACCAACATGGGCCACAACTA | GCCGGAGGGATCTCGAATTT |
| *SlNRT2.2* | NM_001279334.1 | Nitrate transporter 2.2  (NRT2.2) | CCCACAATGGGGAAGCATGT | TTTGCCTCTCTGCCTCTGTG |
| *SlNii1* | NM_001346905.1 | Nitrite reductase  (Nii1) | TTGGCTGATGAGTACGGCTC | GGCTCTTTTAGCAGTGCGTC |
| *SlNiR* | XM_004248688.4 | Ferredoxin-nitrite reductase (NiR) | CGAACTGGGTGTTGAAGGAT | TCCTTTCCCATTGTTTCAGG |
| *SlGS2cp* | NM_001323669.1 | Glutamine synthetase 2  (GS2) | AATTGAGGGTGACTGGAACG | GTGGCGAAGGGATAGATTCA |
| *SlNAR2.1* | XM_010320725.3 | Nitrate transporter 3.2  (NAR2.1) | AGCGGACGACATGCTACTTT | TGCCCTGGCCTTTCTCTTTT |
| *SlGOGAT* | XM_010319707.3 | Glutamate synthase 1 [NADH]  (GOGAT) | CCCTCATAACACTTGGCTGC | TCCAGCAAACTTCTCCCGAA |
| *SlNR* | NM_001328498.1 | Nitrate reductase (NR) | ACCGATGAGACCCTCAACAC | CTCTCCCTTGTGAGGTTTGC |
| *SlEF1* | X14449.1 | Elongation factor 1 alpha  (EF1) | TGGATATGCTCCAGTGCTTG | TTCCTTACCTGAACGCCTGT |
| *SlUbi* | NM_001346406.1 | Ubiquitin (ubi3)  (Ubi) | AGCCAAAGAAGATCAAGCACA | GCCTCTGAACCTTTCCAGTG |
| *SlIRT1* | NM_001247319.1 | Iron-regulated transporter 1 (IRT1) | GCGGACAACATTGCCACAAA | CTGGCTACGGGGTTTATGCA |
| *SlIRT2* | NM_001247323.1 | Iron-regulated transporter 2 (IRT2) | GGGTGCAGTAAATGGTGGACA | TCCCAGCTCTAACACCATAGC |
| *SNRAMP1* | NM_001247389.1 | Root-specific metal transporter (NRAMP1) | ATTGGCCTGCAGAGATATGG | TTCCCCAAAGAAACAAGCTG |
| *SlOPT3* | XM_019211035.2 | Oligopeptide transporter 3 | GAAGCTCTTCATCCGGACAG | AATCCTCCGGGACCAAATAC |
| *SlLHA4* | NM_001324146.1 | H(+)-ATPase4 (LHA4) | TCCTTCGTTGAACGTCCAGG | CTTGCAAAAGTCCAGTCGGC |
| *SlFER(bHLH)* | NM_001247725.2 | bHLH transcriptional regulator (fer) | ACATTGCCAGATCCTATTTCGC | TTTTGGTGGTAGCCGTTGTG |
| *SlFRO1* | NM_001247400.2 | Ferric-chelate reductase (FRO1) | ACTGGGGCTACAAATCGAGG | TCAGATGGGTTGGGCTTGAA |
