# Supplementary material for: A multi-omics insight on the interplay between iron deficiency and N forms in tomato
Source: Front Plant Sci. 2024 Oct 16;15:1408141. doi: 10.3389/fpls.2024.1408141 (PMC11521840; doi:10.3389/fpls.2024.1408141)
Supplement: Supplementary file 4 [file Table3.docx]

**Supplementary Table S3:** Elemental content in tomato young leaves, old leaves and roots**.** Data refers to mean values ± SD; letters refer to statistical significance for each element and plant organ among experimental conditions (+Fe/+Fe-N; -Fe/-Fe-N; -Fe/+Fe+Nit; -Fe/+Fe+U; -Fe+Fe+A; Holm–Sidak test ANOVA. N =3. p-value < 0.05). Data are expressed in μg.

| μg | Cu | Fe | Mn | Na | Zn |
| --- | --- | --- | --- | --- | --- |
|  |  |  |  |  |  |
| *Young leaves* |  |  |  |  |  |
| +Fe/+Fe-N | 3.2±0.3^b^ | 50.1±8.6^a^ | 8.4±0.6^c^ | 53.2±10.1^a^ | 8±0.4^c^ |
| -Fe/-Fe-N | 5.2±0.8^a^ | 17.6±3^c^ | 12.6±1.1^b^ | 24.9±5.2^b^ | 10.7±0.8^b^ |
| -Fe/+Fe+Nit | 5.4±0.8^a^ | 28.3±4.8^b^ | 16.8±1.2^a^ | 33.7±3.6^b^ | 13.3±0.7^a^ |
| -Fe/+Fe+U | 5±0.3^a^ | 17.8±1.2^c^ | 13±1.2^b^ | 30.7±4.2^b^ | 10.9±0.2^b^ |
| -Fe/+Fe+A | 5.9±0.6^a^ | 30.5±4.7^b^ | 13±1.3^b^ | 38.4±2.6^ab^ | 10.9±1.4^b^ |
|  |  |  |  |  |  |
| *Old leaves* |  |  |  |  |  |
| +Fe/+Fe-N | 3.5±0.3^b^ | 105±5.6^a^ | 13.7±0.7^c^ | 190±10.3^a^ | 11.1±0.5^c^ |
| -Fe/-Fe-N | 8.5±1.7^a^ | 27±1.2^c^ | 27.8±4.7^b^ | 126±4.9^c^ | 21.3±3.4^b^ |
| -Fe/+Fe+Nit | 8.5±2.1^a^ | 39±7^b^ | 44.8±3.4^a^ | 148±10.1^b^ | 34.4±4.5^a^ |
| -Fe/+Fe+U | 7.6±1.2^a^ | 26.4±3.2^c^ | 29.1±5.1^b^ | 140±4.3^bc^ | 20±2.3^b^ |
| -Fe/+Fe+A | 10.5±1.4^a^ | 44.3±5.9^b^ | 35.9±3.7^ab^ | 142±4.3^bc^ | 24.9±4.6^b^ |
|  |  |  |  |  |  |
| *Roots* |  |  |  |  |  |
| +Fe/+Fe-N | 11.5±0.4^c^ | 111±9.8^a^ | 11.1±1^b^ | 411±127^a^ | 13.7±0.4^c^ |
| -Fe/-Fe-N | 41.6±10.5^b^ | 5.4±1^e^ | 12±1.9^b^ | 50.3±17.5^b^ | 38.4±11.2^ab^ |
| -Fe/+Fe+Nit | 61.4±9.6^a^ | 42.4±9.8^b^ | 17.6±3.2^a^ | 80.2±16.6^b^ | 51.9±6.7^a^ |
| -Fe/+Fe+U | 31.7±4^b^ | 26.2±3.6^c^ | 7.9±0.8^b^ | 50.7±1.2^b^ | 27.3±5.9^bc^ |
| -Fe/+Fe+A | 28.8±5.2^b^ | 12.1±2.9^d^ | 9.1±1^b^ | 49±3.2^b^ | 31.6±7.6^bc^ |
|  |  |  |  |  |  |
| µg | Ca | K | Mg | P | S |
|  |  |  |  |  |  |
| *Young leaves* |  |  |  |  |  |
| +Fe/+Fe-N | 2826±40.9^b^ | 10201±64.7^ab^ | 1201±48.8^b^ | 1424±69.7^b^ | 4471±429^ab^ |
| -Fe/-Fe-N | 3569±268^ab^ | 9335±968^b^ | 1798±92.4^a^ | 1510±124^ab^ | 4079±592^b^ |
| -Fe/+Fe+Nit | 4172±325^a^ | 11589±537^a^ | 1852±57.5^a^ | 1743±137^a^ | 2782±481^c^ |
| -Fe/+Fe+U | 3827±49.7^ab^ | 8964±428^b^ | 1821±38.2^a^ | 1509±22.6^ab^ | 3535±373^bc^ |
| -Fe/+Fe+A | 3723±713^ab^ | 11045±793^a^ | 1807±182^a^ | 1695±37.9^b^ | 5311±273^a^ |
|  |  |  |  |  |  |
| *Old leaves* |  |  |  |  |  |
| +Fe/+Fe-N | 6988±150^b^ | 16520±773^ab^ | 1919±85.5^b^ | 1850±211^c^ | 15810±346^ab^ |
| -Fe/-Fe-N | 10513±1792^ab^ | 14035±1159^b^ | 3329±466^a^ | 2217±198^c^ | 15531±2842^ab^ |
| -Fe/+Fe+Nit | 14365±828^a^ | 19381±2344^a^ | 3417±249^a^ | 3227±204^a^ | 14776±2043^b^ |
| -Fe/+Fe+U | 11065±628^ab^ | 13662±828.7^b^ | 3303±144^a^ | 2234±101^c^ | 15310±1775^b^ |
| -Fe/+Fe+A | 13322±2808^ab^ | 17300±2824^ab^ | 3605±618^a^ | 2739±142^b^ | 20667±1311^a^ |
|  |  |  |  |  |  |
| *Roots* |  |  |  |  |  |
| +Fe/+Fe-N | 349±47.3 | 5921±1133 | 218±40^b^ | 537±72.4 | 1029±150 |
| -Fe/-Fe-N | 524±114 | 6710±972 | 430±108^b^ | 758±129 | 1178±182 |
| -Fe/+Fe+Nit | 516±97.4 | 5373±552 | 989±274^a^ | 698±29.1 | 997±42.2 |
| -Fe/+Fe+U | 530±46 | 4592±34 | 520±111^b^ | 610±15 | 912±34 |
| -Fe/+Fe+A | 480±147 | 4805±1024 | 245±50.6^b^ | 598±83.6 | 947±173 |
